# Comparing the efficacy of unilateral biportal endoscopic transforaminal lumbar interbody fusion and minimally invasive transforaminal lumbar interbody fusion in lumbar degenerative diseases: a systematic review and meta-analysis

**DOI:** 10.1186/s13018-023-04393-1

**Published:** 2023-11-22

**Authors:** Haopeng Luan, Cong Peng, Kai Liu, Xinghua Song

**Affiliations:** 1 Department of Spine Surgery, The Six Affiliated Hospital of Xinjiang Medical University, Urumqi, Xinjiang China; 2 Department of Trauma and Microreconstructive Surgery, The First Affiliated Hospital of Xinjiang Medical University, Urumqi, Xinjiang China

**Keywords:** Unilateral biportal endoscopic, Transforaminal lumbar interbody fusion, Lumbar degenerative diseases

## Abstract

**Objective:**

To compare the efficacy and safety of unilateral biportal endoscopic transforaminal lumbar interbody fusion (BE-TLIF) and minimally invasive transforaminal lumbar interbody fusion (MIS-TLIF) in lumbar degenerative diseases.

**Methods:**

This study was registered on International Prospective Register of Systematic Reviews (PROSPERO) (ID: CRD42023432460). We searched PubMed, Embase, Web of Science, Cochrane Library, China National Knowledge Infrastructure, Wan Fang Database, and Wei Pu Database by computer to collect controlled clinical studies on the efficacy and safety of unilateral BE-TLIF and MIS-TLIF in lumbar degenerative diseases from database establishment to May 2023. Two researchers screened the literature, extracted data and evaluated the risk of bias of the included studies, recorded the authors, and sample size, and extracted the intraoperative blood loss, operation time, postoperative drainage, Oswestry disability index, Visual analogue scale, lumbar lordosis, disk height, hospital length stay, fusion rate, and complications in each study. Meta-analysis was performed using Revman 5.4 software provided by Cochrane Library.

**Results:**

A total of 14 cohort studies with a total of 1007 patients were included in this study, including 472 patients in the BE-TLIF group and 535 patients in the MIS-TLIF group. The BE-TLIF group had lower intraoperative blood loss than the MIS-TLIF group [mean difference (MD) = − 78.72, 95% CI (− 98.47, − 58.97), *P* < 0.00001] and significantly reduced postoperative drainage than the MIS-TLIF group [MD = − 43.20, 95% CI (− 56.57, − 29.83), *P* < 0.00001], and the operation time was longer than that of the MIS-TLIF group [MD = 22.68, 95% CI (12.03, 33.33), *P* < 0.0001]. Hospital length stay in BE-TLIF group was significantly less than that in MIS-TLIF group [MD = − 1.20, 95% CI (− 1.82, − 0.57), *P* = 0.0002].

**Conclusion:**

Compared with MIS-TLIF, BE-TLIF for lumbar degenerative diseases has the advantages of less intraoperative blood loss, less early postoperative low back and leg pain, shorter postoperative hospital length stay, and faster early functional recovery.

## Introduction

In recent years, minimally invasive transforaminal lumbar interbody fusion (MIS-TLIF) has emerged as a standard surgical technique for minimally invasive lumbar interbody fusion. Its widespread adoption in clinical practice can be attributed to its minimal invasiveness, quick postoperative recovery, and relatively short learning curve [1, 2]. However, MIS-TLIF has its drawbacks. Due to the depth of the surgical site, the distractor blade cannot fully retract all soft tissues. This can allow some tissues to obstruct the surgical view, complicating the procedure. Additionally, the distractor blades can overstretch the paravertebral muscles, causing ischemia, which may hinder postoperative recovery [3]. As spinal endoscopic techniques continue to gain popularity and widespread use, the unilateral biportal endoscopic (UBE) technique has been progressively integrated into clinical practice. Since its visualization and operational components are situated in separate channels, they don't interfere with each other. This provides a broad field of view, making device manipulation easier and ensuring thorough nerve decompression [4]. Heo et al. [5] first applied UBE technique to complete lumbar interbody fusion in 2017 and obtained good clinical results. There is no previous meta-analysis related to the two. The purpose of this study is to analyze and compare the clinical efficacy of unilateral biportal endoscopic transforaminal lumbar interbody fusion (BE-TLIF) and MIS-TLIF in the treatment of lumbar degenerative diseases and to explore a more suitable minimally invasive lumbar fusion.

## Methods

This meta-analysis followed the Cochrane handbook for conducting and the Preferred Reporting Items for Systematic Reviews and Meta-Analysis (PRISMA) guidelines for reporting [6, 7]. Two authors separately conducted literature retrieval, study eligibility, data extraction, and quality assessment with inconsistency solved by discussion and decided by the corresponding author.

### Literature search

We searched PubMed, Embase, Web of Science, Cochrane Library, China National Knowledge Infrastructure (CNKI), Wan Fang Database, and Wei Pu Database by computer to collect controlled clinical studies on the efficacy and safety of unilateral biportal endoscopic transforaminal lumbar interbody fusion (BE-TLIF) and minimally invasive transforaminal lumbar interbody fusion (MIS-TLIF) in lumbar degenerative diseases from database establishment to May 2023. We restricted the language to English and Chinese. By preserving the literature that offered the most comprehensive information for overlapping patients, information duplication was avoided. The brief retrieval formula was “(unilateral biportal endoscopic) AND (lumbar) AND (fusion)”.

### Inclusion and exclusion criteria

The inclusion criteria were as follows: (1) patients treated with BE-TLIF or MIS-TLIF for lumbar degenerative diseases and (2) the literature reported one of the following: intraoperative blood loss, operation time, postoperative drainage, Oswestry disability index (ODI), Visual analogue scale (VAS), lumbar lordosis (LL), disk height (DH), hospital length stay, fusion rate, and complications.

Exclusion criteria were as follows: (1) combined with lumbar infectious diseases, neoplastic diseases or lumbar fractures and other diseases; (2) the index level with a history of previous lumbar spine surgery; (3) review, meeting, expert opinion, case report, literature that could not obtain the full text; and (4) animal experiments and in vitro/biomechanical studies.

### Literature screening and data extraction

Two investigators independently screened the literatures according to the inclusion and exclusion criteria, extracted the data, and cross-checked. In case of any disagreement, the disagreement was discussed and resolved. If necessary, the opinion of the third investigator was solicited, and the information was extracted using a predesigned data extraction form. The main information extracted from the data included: (1) general information about the included studies, including the title, author, publication year, etc.; (2) study characteristics, including the study region, sample size, age, operation time, follow-up time, etc.; (3) outcome measures of interest included intraoperative blood loss, operation time, postoperative drainage, ODI, VAS, LL, DH, hospital length stay, fusion rate and complications; and (4) key elements of bias risk evaluation, including the selection of study population, comparability between groups, and measurement of exposure factors.

### Literature quality evaluation

The risk of bias evaluation of the included literatures was independently completed by two evaluators and cross-checked. If there was disagreement on the evaluation results of the literatures, the third party intervened to assist in the discussion and decision. Cochrane Handbook recommended 5.4 Bias Risk Assessment Tool was used to assess the quality of literatures, including sequence generation, allocation concealment, blinding, data integrity, selective reporting, and other potential biases, and the judgment of deviations was expressed as "low risk", "high risk" or "unclear risk". The Newcastle–Ottawa Scale (NOS) risk bias assessment criteria were used to assess the quality of the cohort study (CS) literature, and articles with a total score of ≥ 7 were regarded as high-quality articles.

### Statistical analysis

Meta-analysis of the data from the included articles was performed using RevMan 5.4 software. Continuous variables were expressed as mean difference (MD) and dichotomous variables as odds ratio (OR), and the size of each pooled effect size and its 95% confidence interval (CI) were calculated. Heterogeneity was analyzed using the Chi-square test, and the size of heterogeneity was judged based on the *I*^2^ value. When *P* > 0.1 or *I*^2^ ≤ 50%, heterogeneity between studies was not significant and fixed effect model was used for analysis; if *P* ≤ 0.1 or *I*^2^ > 50%, heterogeneity between studies was significant, and random effect model was used for analysis.

## Results

### Literature screening procedure and results

**Fig. 1:**
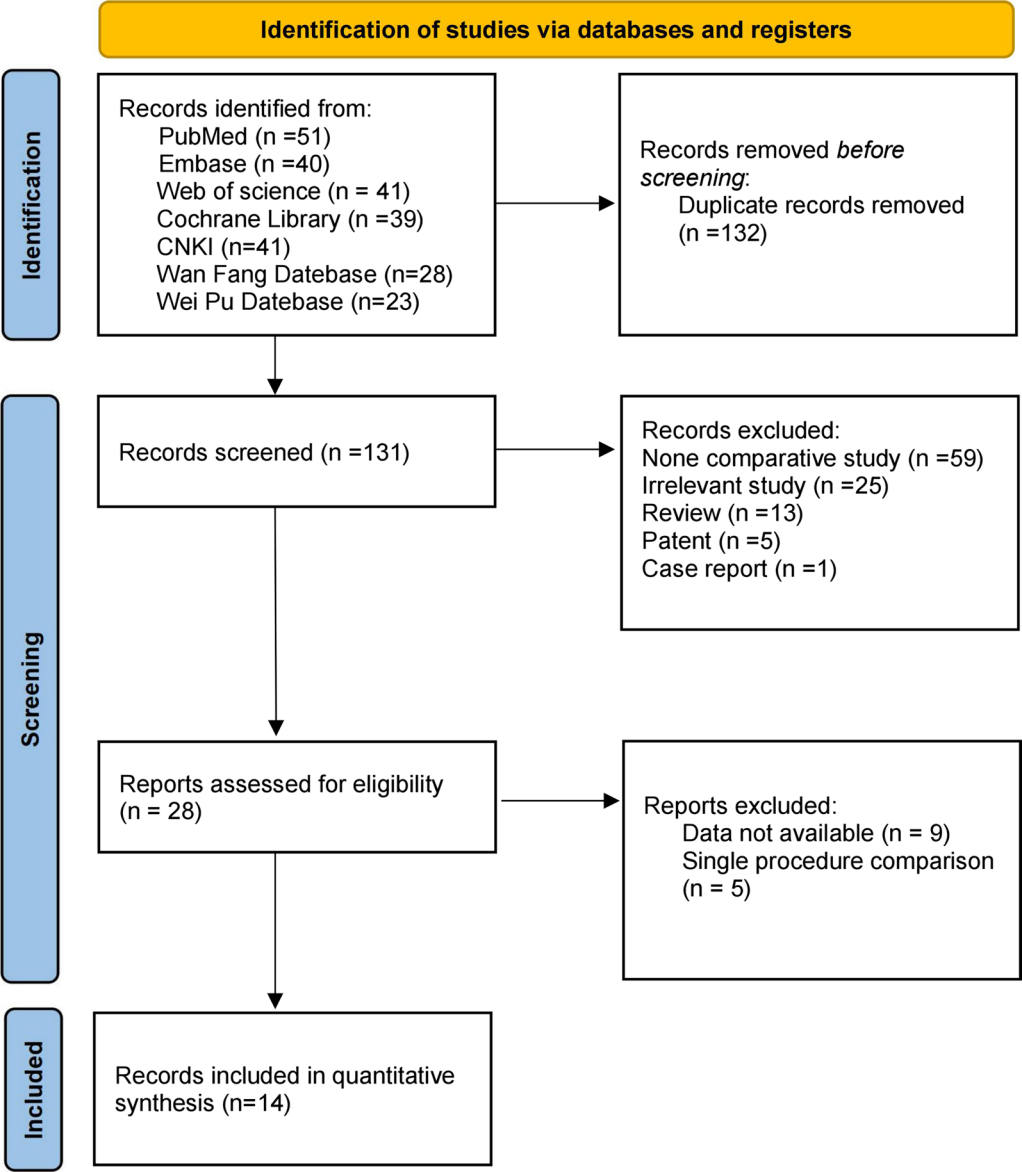
Flowchart illustrating the literature search and the selection of included studies

In this study, 263 papers were obtained through a preliminary search, 132 repeated publications were eliminated by software, titles, and abstracts were read, and 103 papers that obviously did not meet the inclusion criteria were eliminated. After careful reading of the full text and quality evaluation, 14 unqualified papers were further excluded, and 14 qualified papers [3, 8–20] were finally included. The paper screening process is presented in Fig. 1. A total of 1007 patients were included, including 472 patients in the BE-TLIF group and 535 patients in the MIS-TLIF group. The main characteristics of the included studies are presented in Table 1.

**Table 1:** The basic characteristics of the included studies

Study	Study design	Country	Group	n	Age (years)	N(Male/Female)	Operation level (*n*)	Follow-up (Month)	Diagnosis (*n*)	Complication (*n*)
Heo [15]	Retrospective	Korea	BE-TLIF	23	61.4 ± 9.4	7/16	L3-4(3);L4-5(17); L5-S1(3)	13.4 ± 2.5	Degenerative spondylolisthesis; Isthmic spondylolisthesis; Central stenosis with instability; Central stenosis with concomitant foraminal stenosis(23)	Epidural hematoma(1); Cage subsidence(1)
			MIS-TLIF	46	63.5 ± 10.5	19/27	L3-4(4);L4-5(29); L5-S1(13)	13.4 ± 2.5	Degenerative spondylolisthesis; Isthmic spondylolisthesis; Central stenosis with instability; Central stenosis with concomitant foraminal stenosis(46)	Dural tear(1); Epidural hematoma(1); Superficial wound infection(1); Deep vein Thrombosis(1); Cage subsidence(2)
Kim [21]	Retrospective	Korea	BE-TLIF	32	70.5 ± 8.26	17/15	L2-3(1);L3-4(3); L4-5(20);L5-S1(8)	27.2 ± 5.4	Degenerative spondylolisthesis(26); Isthmic spondylolisthesis(6)	Epidural hematoma(1); Transient palsy(1)
			MIS-TLIF	55	67.3 ± 10.7	25/30	L3-4(2);L4-5(46); L5-S1(7)	31.5 ± 7.3	Degenerative spondylolisthesis(48); Isthmic spondylolisthesis(7)	Epidural hematoma(1); Transient palsy(2)
Kang [8]	Retrospective	Korea	BE-TLIF	47	66.87 ± 10.41	17/30	L2-3(4);L3-4(7); L4-5(34);L5-S1(20)	14.5 ± 2.3	Lumbar spinal stenosis; Spondylolisthesis(47)	Incomplete decompression(1); Hematoma(2); Dural tear(3)
			MIS-TLIF	32	66.38 ± 9.45	17/15	L2-3(1);L3-4(9); L4-5(22);L5-S1(11)	15.78 ± 3.16	Lumbar spinal stenosis; Spondylolisthesis(32)	Incomplete decompression(2); Hematoma(1); Dural tear(1);Infection (1)
Gatam [16]	Retrospective	Indonesia	BE-TLIF	72	55.1 ± 5.12	26/46	L3-4(8);L4-5(56); L5-S1(8)	12	Single level degenerative spondylolisthesis(72)	Dural tear (3)
			MIS-TLIF	73	52.3 ± 6.13	28/45	L3-4(10);L4-5(48); L5-S1(15)	12	Single level degenerative spondylolisthesis(73)	Postoperative infection(2); Cage subsidence(2)
Zhu [14]	Retrospective	China	BE-TLIF	35	50.94 ± 12.12	16/19	L4-5(28);L5-S1(7)	15.29 ± 1.98	Lumbar spinal stenosis(19);Lumbar disk herniation(7);Spondylolisthesis(9)	Transient lower extremity numbness(2)
			MIS-TLIF	41	53.44 ± 14.37	19/22	L3-4(2);L4-5(25); L5-S1(14)	16.12 ± 2.59	Lumbar spinal stenosis(21);Lumbar disk herniation(13);Spondylolisthesis(7)	Transient lower extremity numbness(2);Epidural hematoma(1)
Ni [20]	Retrospective	China	BE-TLIF	27	50.4 ± 11.4	9/18	L2-3(2);L3-4(0);L4-5(18);L5-S1(7)	13.3 ± 1.0	Lumbar disk herniation with instability(15); Lumbar spinal stenosis with instability(12)	No complications
			MIS-TLIF	33	53.4 ± 13.5	15/18	L2-3(0);L3-4(3);L4-5(21);L5-S1(9)	13.4 ± 1.2	Lumbar disk herniation with instability(21); Lumbar spinal stenosis with instability(12)	No complications
Jiang [19]	Retrospective	China	BE-TLIF	25	63.28 ± 8.51	9/16	L4-5(24);L5-S1(1)	NR	Single-segment lumbar stenosis with instability(25)	No complications
			MIS-TLIF	25	59.68 ± 10.38	8/17	L4-5(23);L5-S1(2)	NR	Single-segment lumbar stenosis with instability(25)	No complications
Song [12]	Retrospective	China	BE-TLIF	28	54.7 ± 10.0	10/18	L3-4(1);L4-5(22); L5-S1(5)	14.1 ± 1.5	Meyerding ISpondylolisthesis(28)	Slight decrease in muscle strength(1)
			MIS-TLIF	28	56.3 ± 11.6	8/20	L3-4(1);L4-5(20); L5-S1(7)	14.3 ± 1.4	Meyerding ISpondylolisthesis(28)	Transient lower extremity numbness(1)
Kong [11]	Retrospective	China	BE-TLIF	35	55.1(39–70)	13/22	L2-3(1);L3-4(5); L4-5(17);L5-S1(10); L4-S1(2)	14.7 ± 2.5	Lumbar disk herniation with spinal stenosis(15); Lumbar spinal stenosis(12); Lumbar spinal stenosis with mild spondylolisthesis(8)	Dural tear(1); Postoperative epidural hematoma(1)
			MIS-TLIF	40	56.0(41–73)	18/22	L1-2(1);L2-3(4); L3-4(7);L4-5(15); L5-S1(12); L4-S1(1)	15.0 ± 3.4	Lumbar disk herniation with spinal stenosis(9); Lumbar spinal stenosis(15); Lumbar spinal stenosis with mild spondylolisthesis(16)	Dural tear(1); Postoperative epidural hematoma (1); Infection(1)
Song [9]	Retrospective	China	BE-TLIF	25	52.36 ± 10.69	9/16	L3-4(2);L4-5(13); L5-S1(10)	14.04 ± 1.51	Lumbar spinal stenosis; Lumbar disk herniation; Spondylolisthesis(25)	Transient lower extremity numbness(1)
			MIS-TLIF	24	56.38 ± 10.53	8/16	L3-4(2);L4-5(10); L5-S1(12)	14.79 ± 1.59	Lumbar spinal stenosis;Lumbar disk herniation; Spondylolisthesis(24)	Transient lower extremity numbness(4)
Huang [10]	Retrospective	China	BE-TLIF	38	60.13 ± 7.36	22/16	L4-5(28);L5-S1(10)	NR	Degenerative Spondylolisthesis(19); Central stenosis with segmental Instability(5); Isthmic Spondylolisthesis(4); Lumbar disk herniation with spinal stenosis(10)	Dural tear(2)
			MIS-TLIF	44	59.68 ± 6.94	26/18	L3-4(1); L4-5(30);L5-S1(13)	NR	Degenerative Spondylolisthesis(22); Central stenosis with segmental Instability(7); Isthmic Spondylolisthesis(6); Lumbar disk herniation with spinal stenosis(9)	Transient neurologic symptom(2)
Heo [17]	Retrospective	Korea	BE-TLIF	32	65.2 ± 19.5	10/22	L4-5(27);L5-S1(5)	13.9 ± 2.6	Degenerative spondylolisthesis(20); Isthmic spondylolisthesis(5); Central stenosis(4); Foraminal stenosis(3)	Postoperative epidural hematoma(2);Pneumonia (1);Transient neurologic symptoms(1);Postoperative ileus(1);Dura tear(1); Wound dehiscence (1); Cage subsidence(4)
			MIS-TLIF	41	62.3 ± 10.6	16/25	L4-5(30);L5-S1(11)	15.0 ± 3.3	Degenerative spondylolisthesis(27); Isthmic spondylolisthesis(5); Central stenosis(5); Foraminal stenosis(2); Recurrent disk herniation(2)	Postoperative epidural hematoma(1);Transient neurologic symptom(1); Cage subsidence(1)
Yu [18]	Retrospective	China	BE-TLIF	23	60.8(45–74)	11/12	L3-4(3);L4-5(11);L5-S1(9)	38.96 ± 3.17	Degenerative spondylolisthesis(16); Isthmic spondylolisthesis(7)	Cage subsidence(2); Dural tear(1)
			MIS-TLIF	18	60.7(46–71)	8/10	L3-4(3);L4-5(8);L5-S1(7)	39.50 ± 3.35	Degenerative spondylolisthesis(14); Isthmic spondylolisthesis(4)	Dural tear(2); Cage subsidence(1); Postoperative infection(1)
Yang [13]	Retrospective	China	BE-TLIF	30	49.3 ± 3.5	12/18	L3-4(6);L4-5(15);L5-S1(9)	NR	Single-level lumbar spinal stenosis; Single-level lumbar disk herniation(30)	Dural tear(1); Transient neurologic symptom(1)
			MIS-TLIF	35	50.9 ± 3.6	20/15	L3-4(7);L4-5(21);L5-S1(7)	NR	Single-level lumbar spinal stenosis; Single-level lumbar disk herniation(35)	Transient neurologic symptom(1); Postoperative epidural hematoma(1)

BE-TLIF = unilateral biportal endoscopic transforaminal lumbar interbody fusion; MIS-TLIF = minimally invasive transforaminal lumbar interbody fusion; NR = not reported

### Quality analysis of included studies

**Fig. 2:**
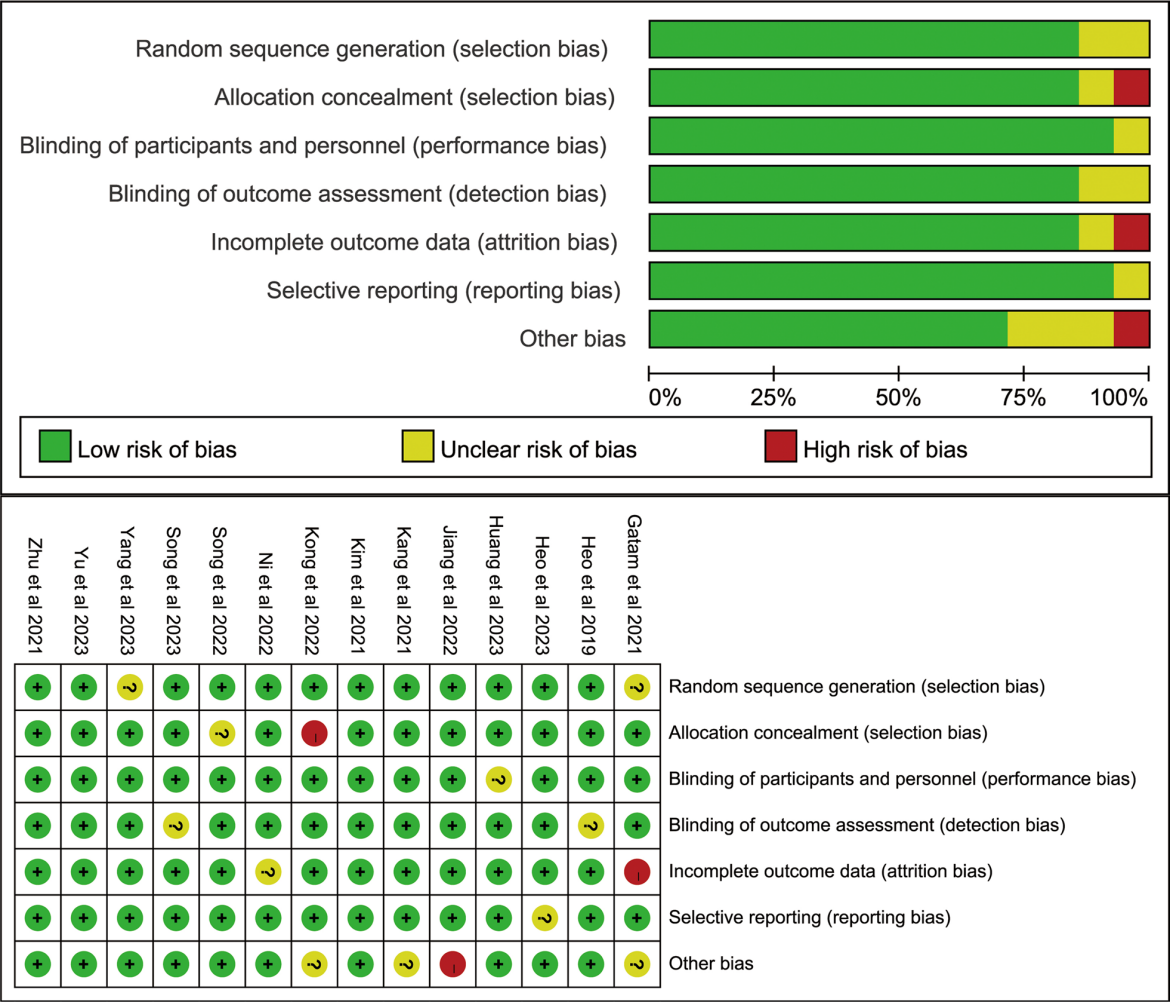
Risk of bias graph for each included study

Risk assessment for the 14 cohort studies included in the analysis was conducted using the Cochrane Risk of Bias tool and is presented in Fig. 2. The quality of non-randomized controlled trials was assessed using the Newcastle–Ottawa Scale (NOS). All included studies scored between 7 and 9 points, indicating high quality. Table 2 provides a summary of the quality scores for each study.

**Table 2:** Quality Assessment Using the Newcastle–Ottawa Quality Assessment Scale for Each Non-Randomized Controlled Trial

Variable	Heo [15]	Kim [21]	Kang [8]	Gatam [16]	Zhu [14]	Ni [20]	Jiang [19]	Song [12]	Kong [11]	Song [9]	Huang [10]	Heo [17]	Yu [18]	Yang [13]
*Selection*														
Representativeness of exposed cohort	1	1	1	1	1	1	1	1	1	1	1	1	1	1
Selection of non-exposed cohort	1	1	1	1	1	1	1	1	1	1	1	1	1	1
Ascertainment of exposure	1	1	1	1	1	1	1	1	1	1	1	1	1	1
Demonstration that outcome of interest was not present at start of study	1			1			1	1			1	1	1	
*Comparability*														
Study controlled for age or gender	1	1	1	1	1	1	1	1	1	1	1	1	1	1
Study controlled for any additional factor	1	1	1					1	1	1		1		1
*Outcome*														
Assessment of outcome	1	1	1	1	1	1	1	1	1	1	1	1	1	1
Follow-up long enough for outcomes to occur	1	1	1	1	1	1	1	1	1	1	1	1	1	1
Adequacy of follow-up of cohort	1	1	1	1	1	1	1	1	1	1	1	1	1	1
Total	9	8	8	8	7	7	8	9	8	8	8	9	8	8

### Meta-analysis results

#### Operation time

**Fig. 3:**
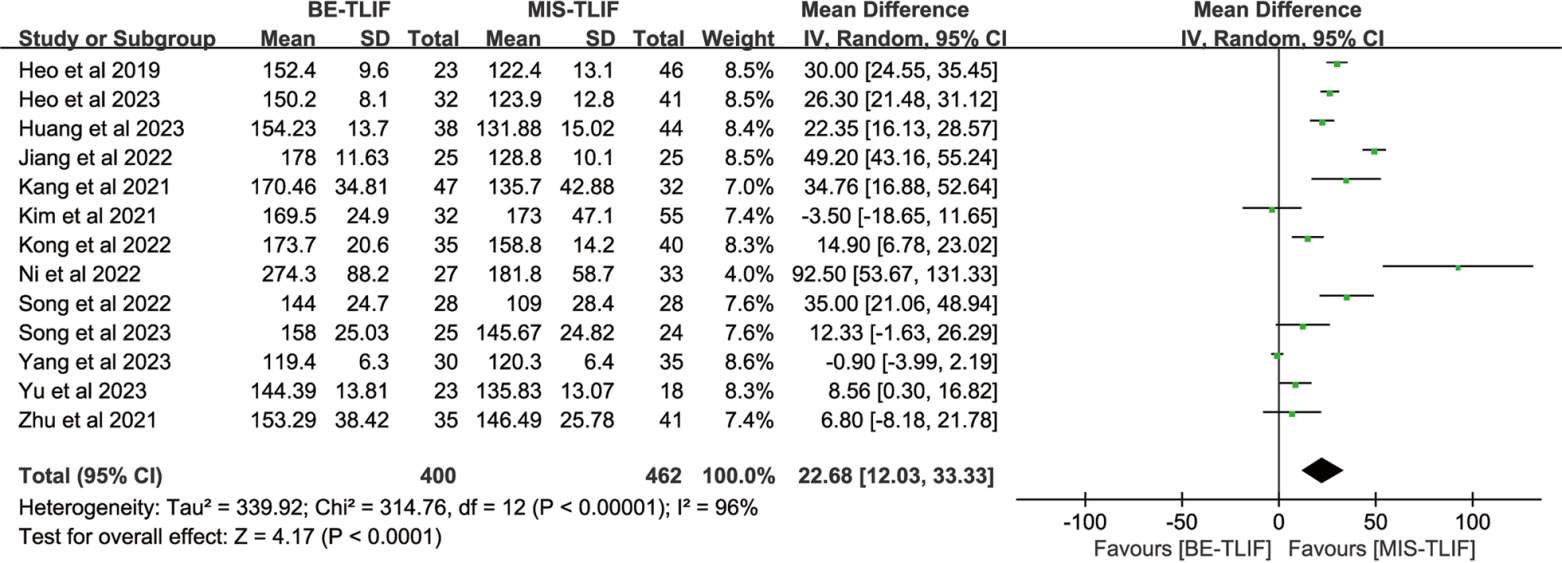
Forest plot of operation time

A total of 13 studies used operation time as an outcome measure, with 400 patients in the BE-TLIF group and 462 patients in the MIS-TLIF group. The heterogeneity test (*P* < 0.00001, *I*^2^ = 96%) suggested that there was significant heterogeneity between the studies, and a meta-analysis using a random-effects model showed that: [MD = 22.68, 95% CI (12.03, 33.33), *P* < 0.0001] (Fig. 3), The results showed that the operation time was longer in BE-TLIF compared to MIS-TLIF.

#### Intraoperative blood loss

**Fig. 4:**
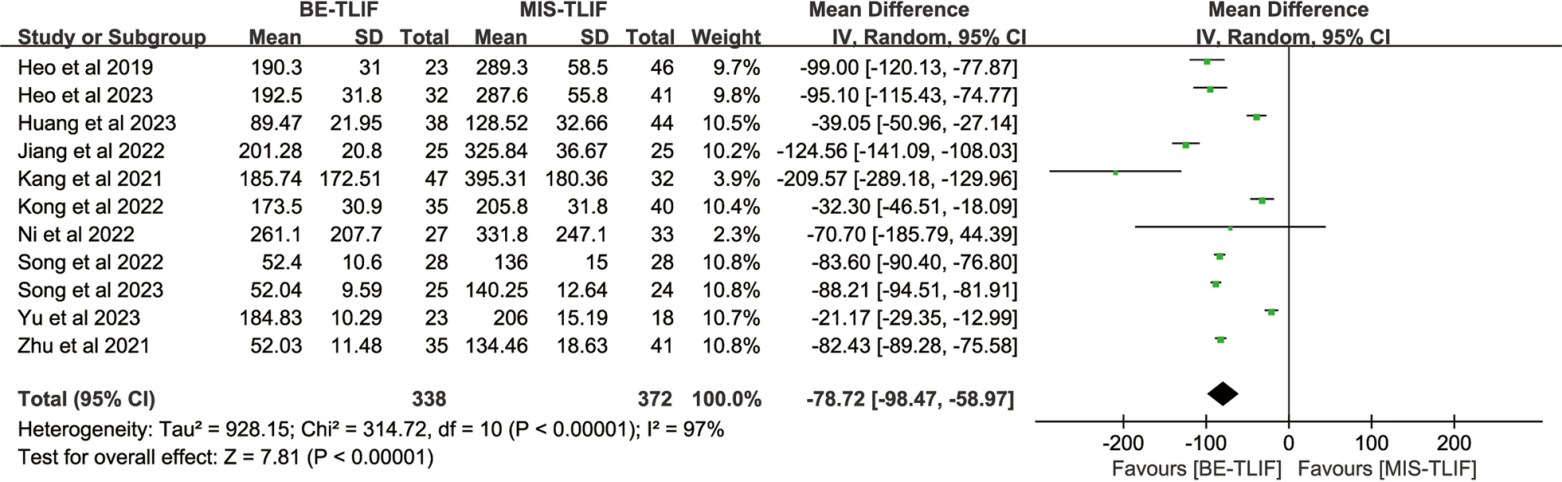
Forest plot of intraoperative blood loss

Intraoperative blood loss was counted in 11 studies, with 338 patients in the BE-TLIF group and 372 patients in the MIS-TLIF group. The heterogeneity test (*P* < 0.00001, *I*^2^ = 97%) suggested that there was significant heterogeneity between the studies. The results showed that intraoperative blood loss in the BE-TLIF group was significantly lower than that in the MIS-TLIF group [MD = − 78.72, 95% CI (− 98.47, − 58.97), *P* < 0.00001] (Fig. 4), indicating that BE-TLIF surgical approach had a certain effect on the reduction of intraoperative blood loss in patients.

#### Postoperative drainage

**Fig. 5:**
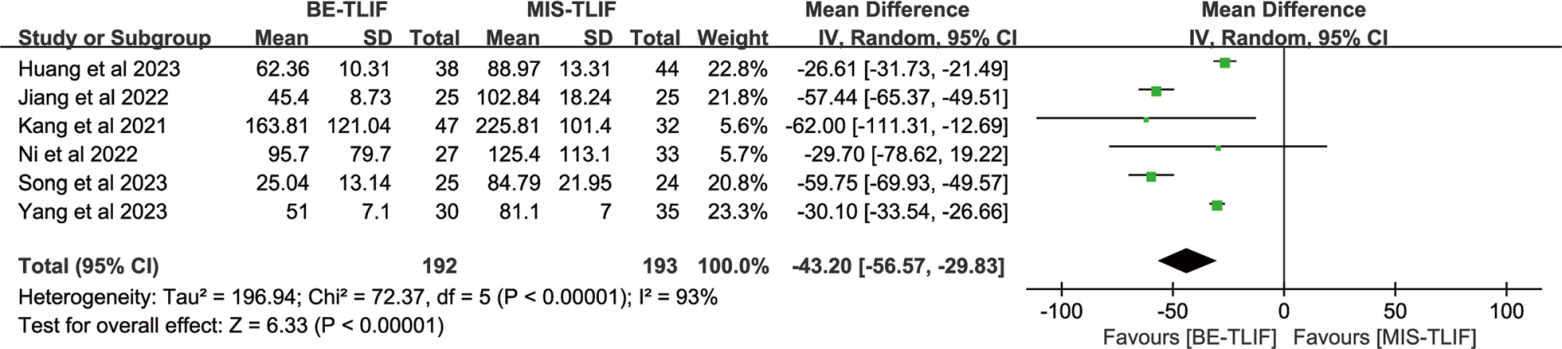
Forest plot of postoperative drainage

Postoperative drainage was reported in six papers, heterogeneity test result *P* < 0.00001; *I*^2^ = 93%. There was significant heterogeneity across the studies. The results showed that postoperative drainage in BE-TLIF group was significantly less than that in MIS-TLIF group [MD = − 43.20, 95% CI (− 56.57, − 29.83), *P* < 0.00001] (Fig. 5).

#### Pain evaluation

Preoperative back VAS scores were reported in 11 papers, and heterogeneity test results showed *P* = 0.90; *I*^2^ = 0%. The results showed that preoperative back VAS scores in BE-TLIF group was significantly lower than that in MIS-TLIF group [MD = − 0.14, 95% CI (− 0.28, − 0.00), *P* = 0.04]. The baseline was inconsistent, so postoperative back VAS was not comparable. After remove the study by Kong et al. [11] there was no significant difference in mean preoperative back VAS between the two groups [MD = − 0.10, 95% CI (− 0.25, 0.04), *P* = 0.17], and there was no heterogeneity between studies (*P* = 0.93; *I*^2^ = 0%). So, only 10 studies were included for comparison. Preoperative leg VAS scores were reported in 12 papers, and heterogeneity test results showed *P* = 0.83; *I*^2^ = 0%. The results showed that there was no significant difference in preoperative leg VAS score between BE-TLIF and MIS-TLIF group [MD = − 0.01, 95% CI (− 0.15, 0.12), *P* = 0.86].

**Fig. 6:**
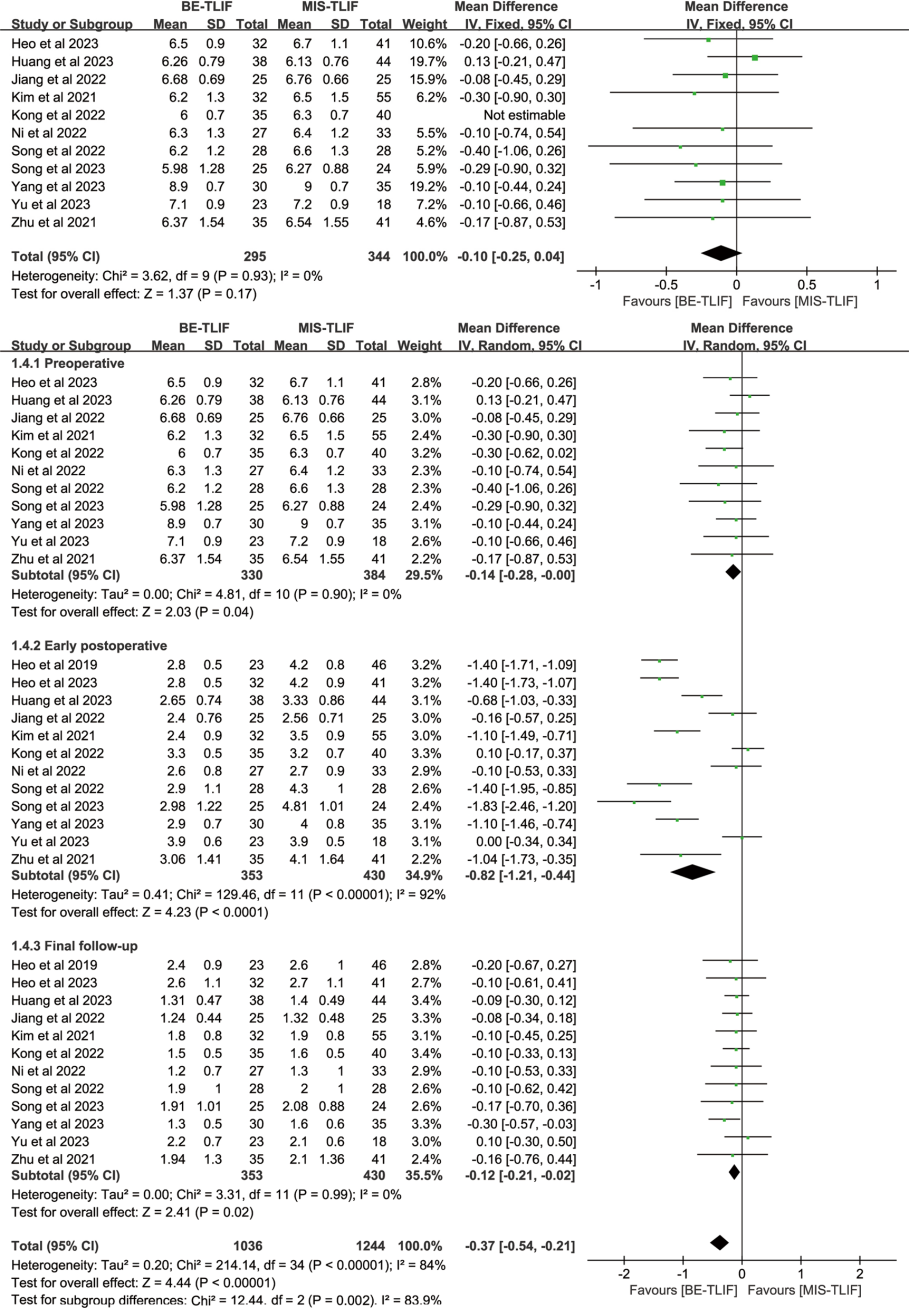
Forest plot of back VAS

**Fig. 7:**
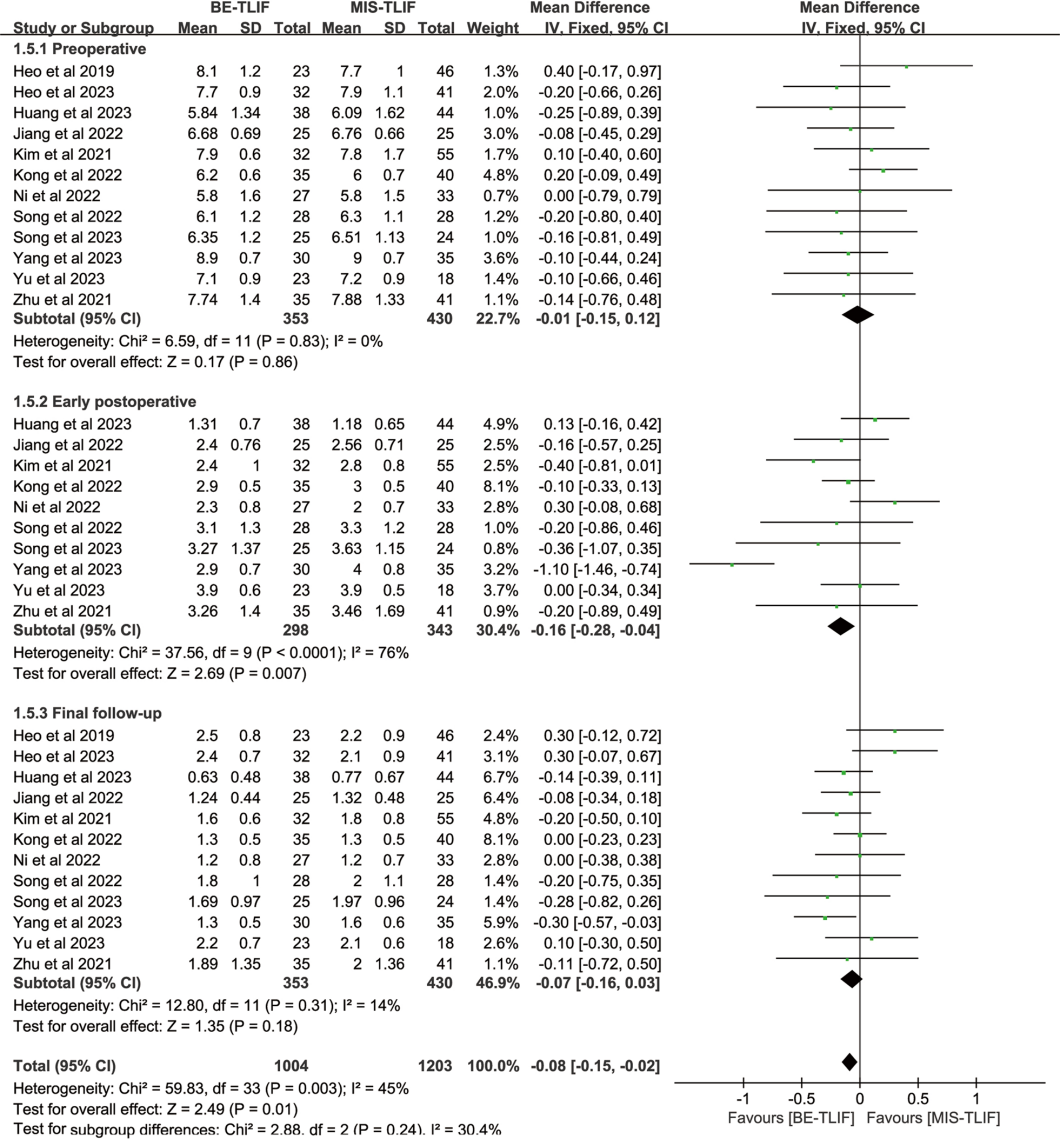
Forest plot of leg VAS

Back VAS scores at early postoperative were reported in 12 papers, and heterogeneity test results showed *P* < 0.00001; *I*^2^ = 92%. The results showed that back VAS score at early postoperative in BE-TLIF group was significantly lower than that in MIS-TLIF group [MD = − 0.82, 95% CI (− 1.21, − 0.44), *P* < 0.0001]. Leg VAS scores at early postoperative were reported in 10 papers, and heterogeneity test results showed *P* < 0.0001; *I*^2^ = 76%. The results showed that leg VAS score at early postoperative in BE-TLIF group was significantly lower than that in MIS-TLIF group [MD = − 0.16, 95% CI (− 0.28, − 0.04), *P* = 0.007] (Fig. 6, Fig. 7).

#### Oswestry disability index

Preoperative Oswestry disability index was reported in 12 papers, and heterogeneity test results showed *P* = 0.11; *I*^2^ = 35%. The results showed that there was no significant difference in the preoperative Oswestry disability index between BE-TLIF and MIS-TLIF group [MD = − 0.52, 95% CI (− 1.61, 0.56), *P* = 0.34].

**Fig. 8:**
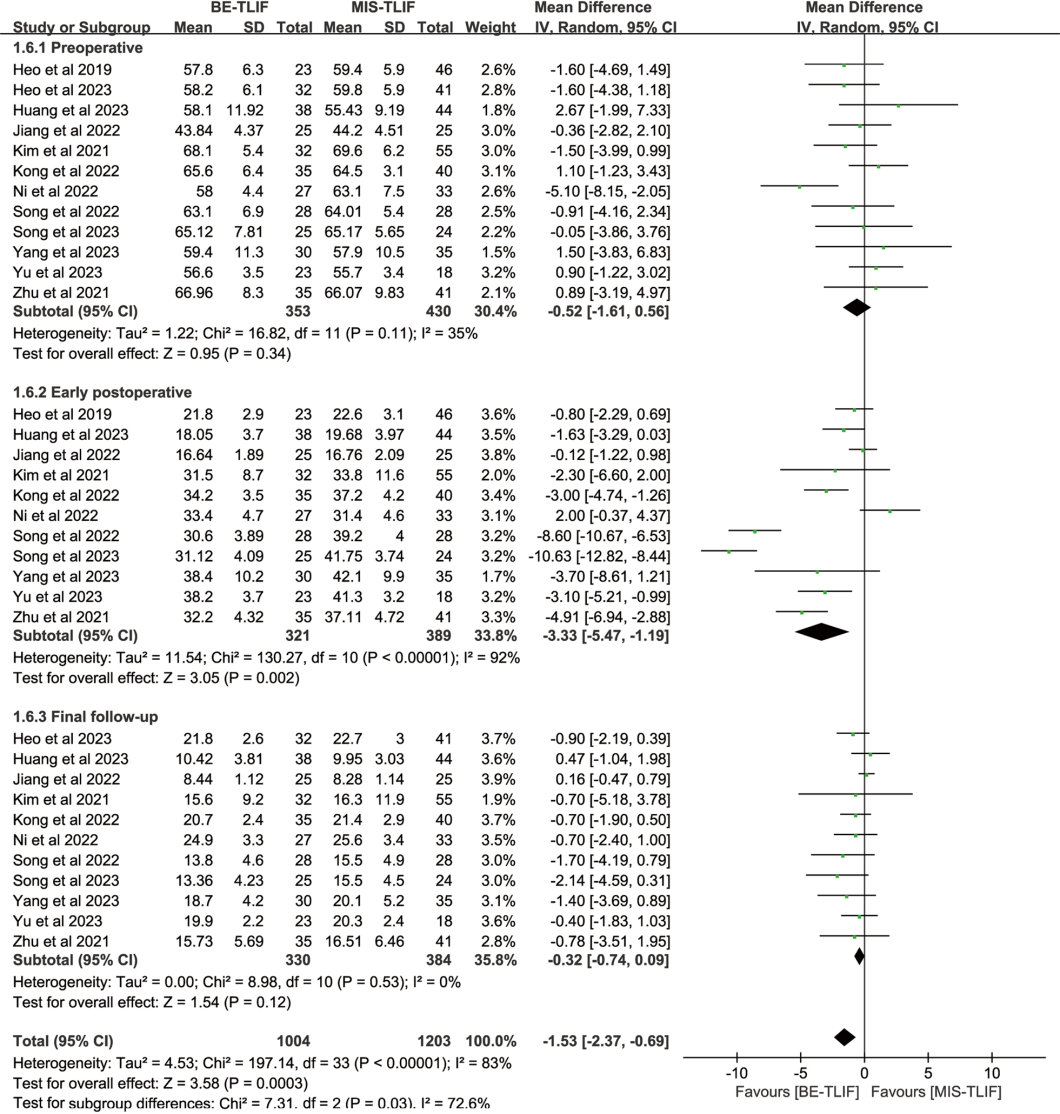
Forest plot of ODI

Postoperative Oswestry disability index was reported in 11 papers, and heterogeneity test results showed *P* < 0.00001; *I*^2^ = 92%. The results showed that back Oswestry disability index at early postoperative in BE-TLIF group was significantly lower than that in MIS-TLIF group [MD = − 3.33, 95% CI (− 5.47, − 1.19), *P* = 0.002] (Fig. 8).

#### Lumbar lordosis

Preoperative lumbar lordosis was reported in 4 papers, and heterogeneity test results showed *P* = 0.79; *I*^2^ = 0%. The results showed that there was no significant difference in preoperative lumbar lordosis between BE-TLIF and MIS-TLIF group [MD = 0.15, 95% CI (− 0.79, 1.09), *P* = 0.76].

**Fig. 9:**
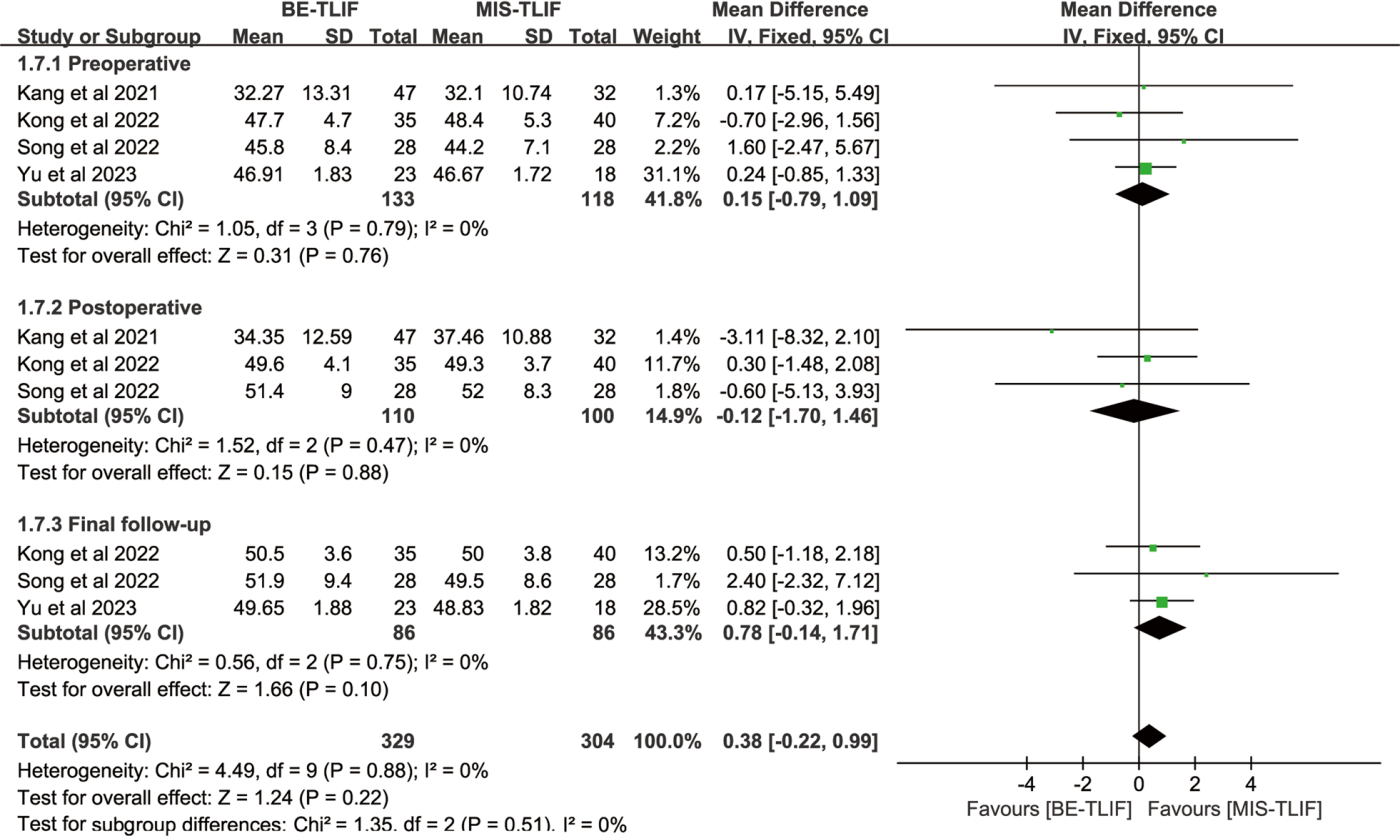
Forest plot of LL

Postoperative lumbar lordosis was reported in three papers, and heterogeneity test results showed *P* = 0.47; *I*^2^ = 0%. The results showed that there was no significant difference in postoperative lumbar lordosis between BE-TLIF and MIS-TLIF group [MD = − 0.12, 95% CI (− 1.70 1.46), *P* = 0.88] (Fig. 9).

#### Disk height

Preoperative disk height was reported in four papers, and heterogeneity test results showed *P* < 0.00001; *I*^2^ = 89%. The results showed that there was no significant difference in preoperative disk height between BE-TLIF and MIS-TLIF group [MD = − 0.34, 95% CI (− 1.52, 0.84), *P* = 0.57].

**Fig. 10:**
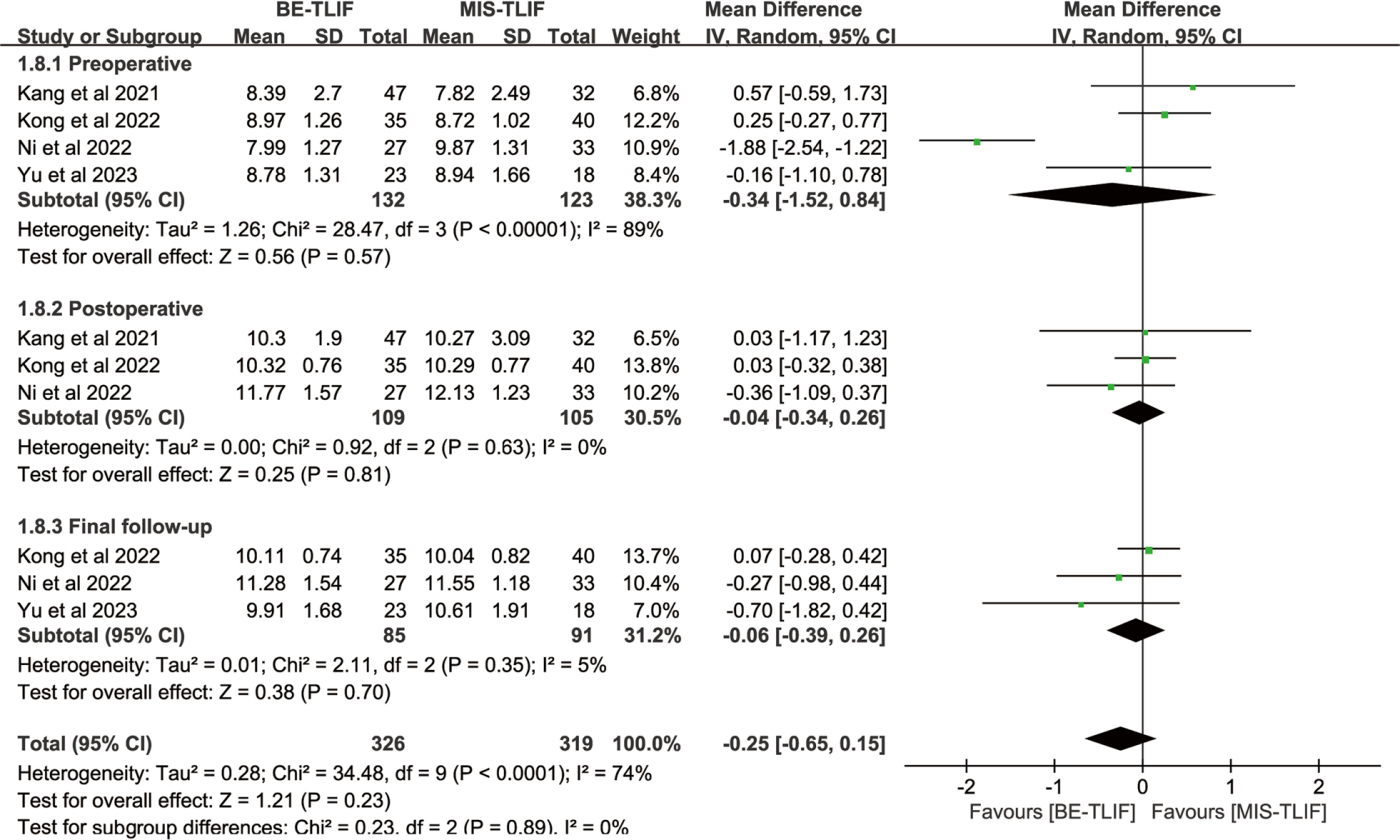
Forest plot of DH

Postoperative disk height was reported in three papers, and heterogeneity test results showed *P* = 0.63; *I*^2^ = 0%. The results showed that there was no significant difference in postoperative disk height between BE-TLIF and MIS-TLIF group [MD = − 0.04, 95% CI (− 0.34 0.26), *P* = 0.81] (Fig. 10).

#### Fusion rate

**Fig. 11:**
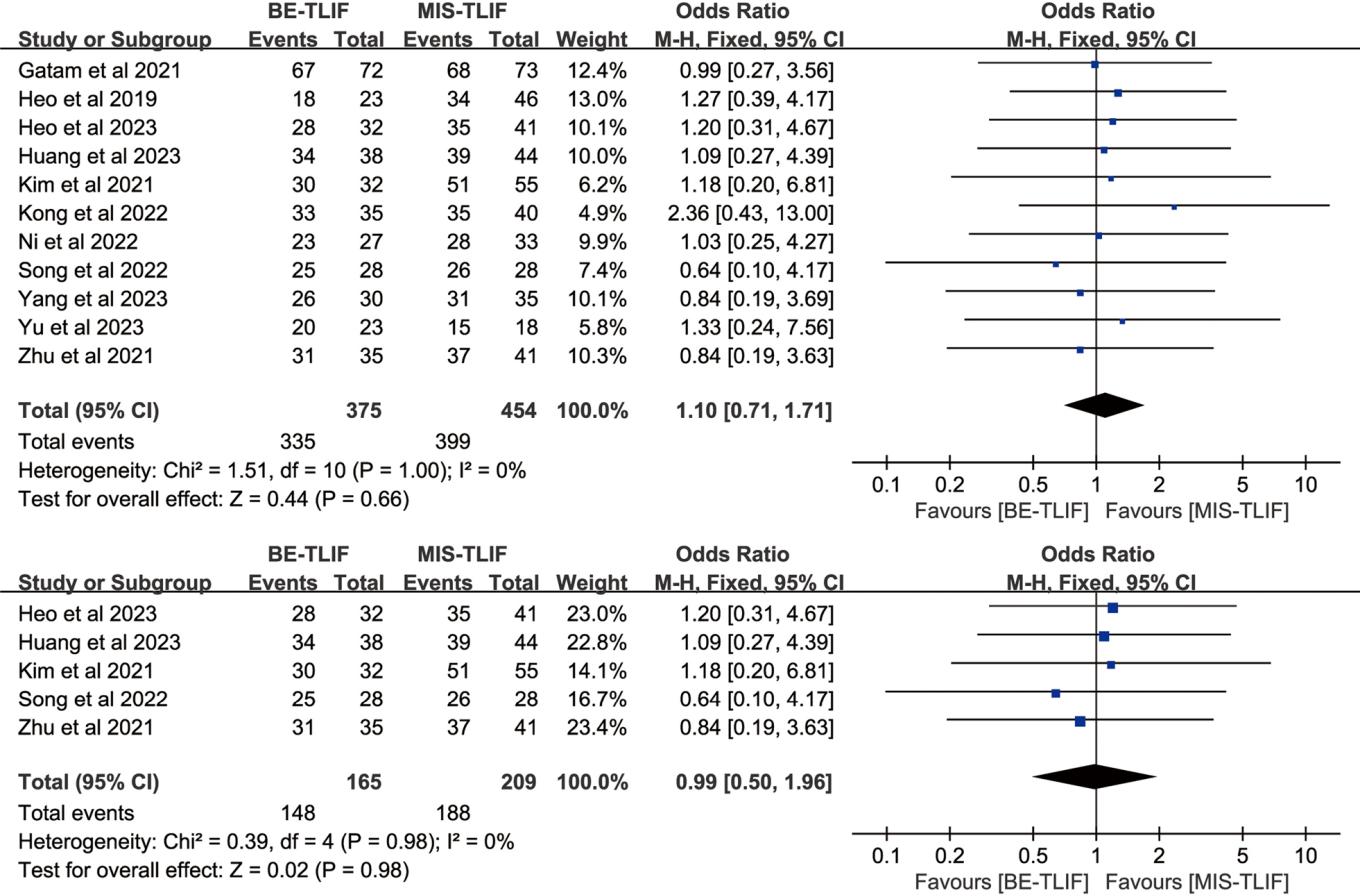
Forest plot of fusion rate

Fusion rate were reported in 11 papers, and heterogeneity test results showed *P* = 1.00; *I*^2^ = 0%. The results showed that there was no significant difference in fusion rate between BE-TLIF and MIS-TLIF group [OR = 1.10, 95% CI (0.71, 1.71), *P* = 0.66]. Five of the 11 articles used Bridwell grading [21] to assess fusion rates, three did not describe the method of assessment, and the remaining three used Suk grading [22], Eck grading [23], Brantigan and Steffee criteria [24] to assess fusion rates, respectively (Fig. 11). Bridwell grading was used in 5 articles, and heterogeneity test results showed *P* = 0.98; *I*^2^ = 0%. The results showed that there was no significant difference in fusion rate between BE-TLIF and MIS-TLIF group [OR = 0.99, 95% CI (0.50, 1.96), *P* = 0.98].

#### Hospital length stay

**Fig. 12:**
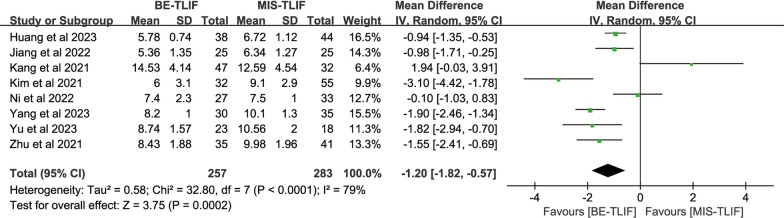
Forest plot of hospital length stay

Hospital length stay was reported in eight papers, heterogeneity test result *P* < 0.0001; *I*^2^ = 79%. There was significant heterogeneity across the studies. The results showed that hospital length stay in BE-TLIF group was significantly less than that in MIS-TLIF group [MD = − 1.20, 95% CI (− 1.82, − 0.57), *P* = 0.0002] (Fig. 12).

#### Complications

**Fig. 13:**
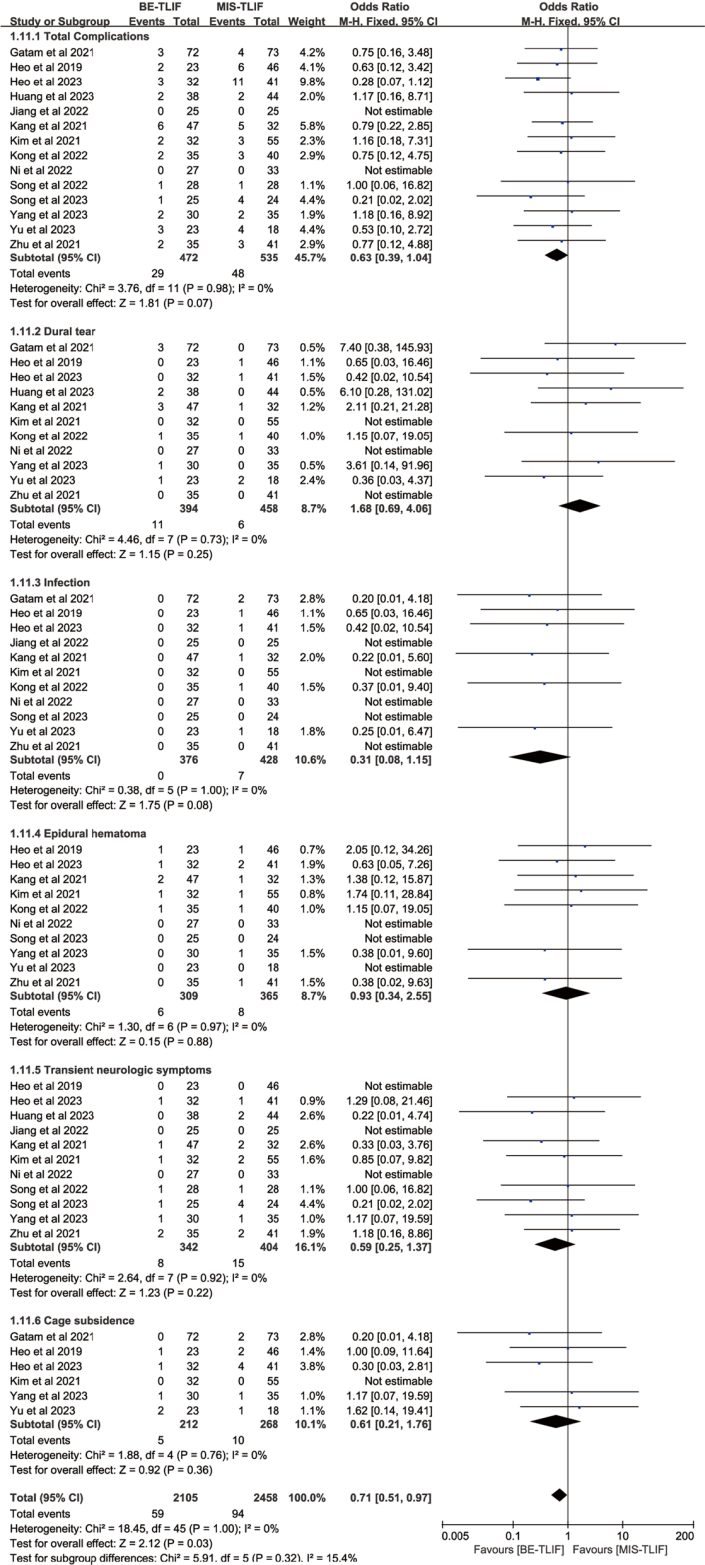
Forest plot of the number of complications

Total complications were reported in 14 papers, and heterogeneity test results showed *P* = 0.98; *I*^2^ = 0%. The results showed that there was no significant difference in total complications between BE-TLIF and MIS-TLIF group [OR = 0.63, 95% CI (0.39, 1.04), *P* = 0.07]. Dural tears were reported in 11 papers, and heterogeneity test results showed *P* = 0.73; *I*^2^ = 0%. The results showed that there was no significant difference in dural tears between BE-TLIF and MIS-TLIF group [OR = 1.68, 95% CI (0.69, 4.06), *P* = 0.25]. Transient neurologic symptoms were reported in 11 papers, and heterogeneity test results showed *P* = 0.92; *I*^2^ = 0%. The results showed that there was no significant difference in transient neurologic symptoms between BE-TLIF and MIS-TLIF group [OR = 0.59, 95% CI (0.25, 1.37), *P* = 0.22]. Among the references included in this article, 65.5% of the patients presented with dural tear and transient neurological symptoms in the BE-TLIF group and 43.7% of the patients presented with dural tear and transient neurological symptoms in the MIS-TLIF group, which accounted for 51.9% of the patients with overall complications in both groups (Fig. 13).

### Heterogeneity and sensitivity analysis

The analysis revealed high heterogeneity in intraoperative blood loss, postoperative drainage and operation time. To assess the impact of heterogeneity on the results, a sensitivity analysis was conducted by removing individual studies from the analysis of operation time. The findings remained consistent with the original conclusions, suggesting that heterogeneity had minimal influence on the study outcomes. Factors contributing to heterogeneity may include differences in surgeon experience, operating techniques, methods for measuring intraoperative blood loss, and completeness and accuracy of case records.

### Publication deviation

The study included 14 articles and tested all outcome measures for publication bias. The funnel plot was visually assessed for each outcome measure, and it appeared to be mostly symmetrical, indicating a low likelihood of publication bias. Figures 14, 15, 16, 17, 18, 19, 20, 21, 22, 23, 24 provide supporting evidence for this finding.

**Fig. 14:**
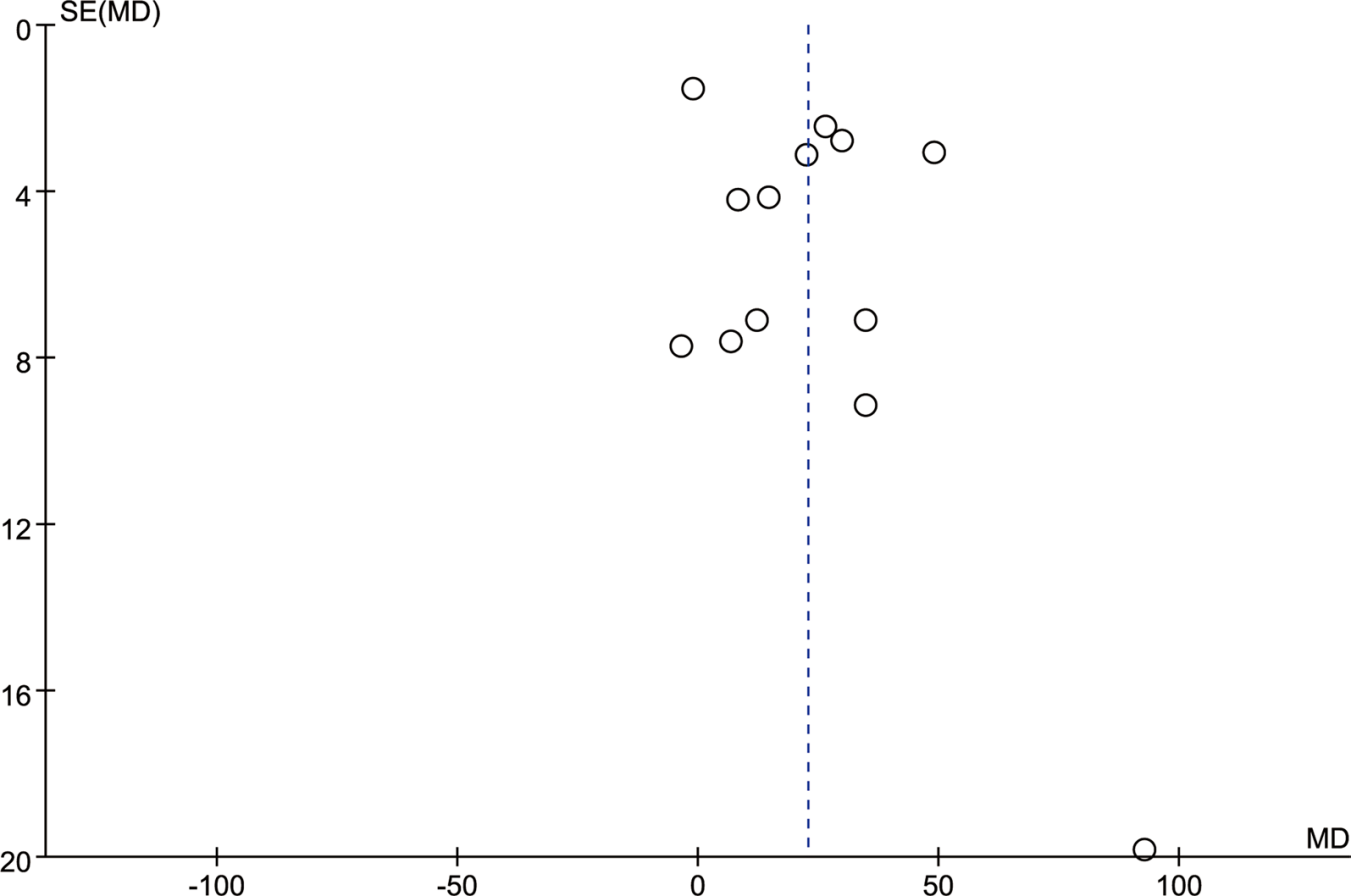
Funnel plot of publication bias for operation time

**Fig. 15:**
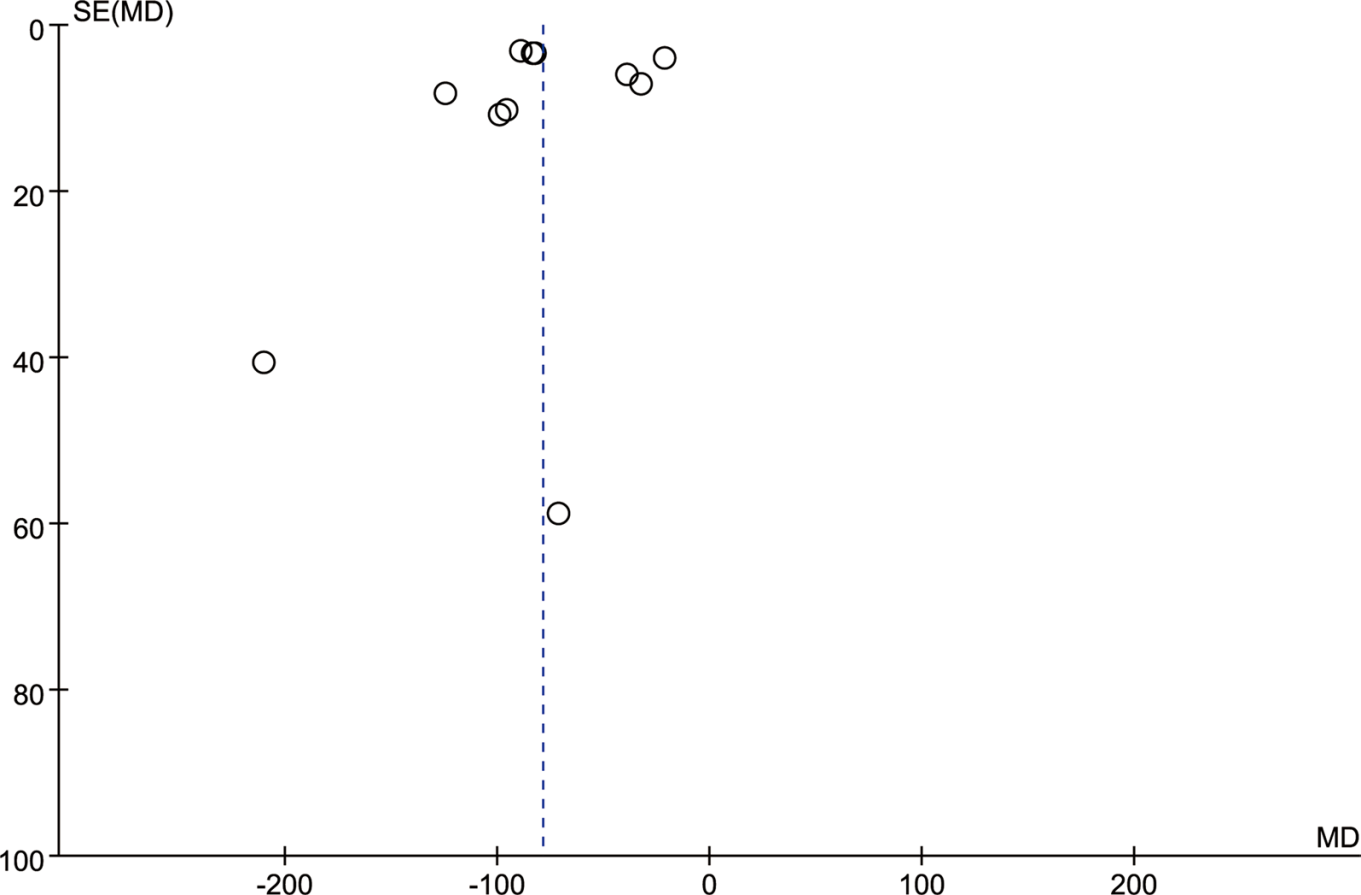
Funnel plot of publication bias for intraoperative blood loss

**Fig. 16:**
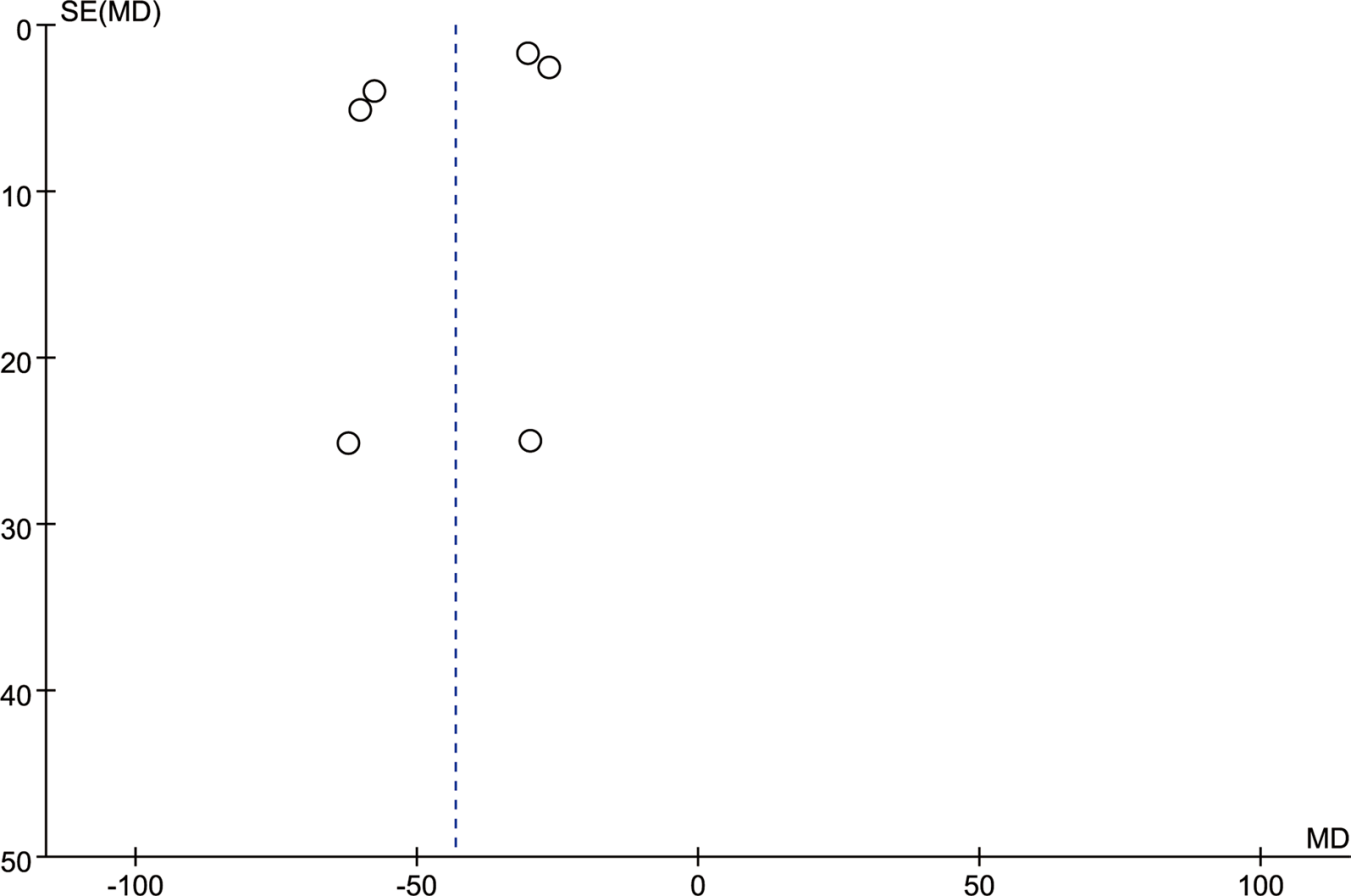
Funnel plot of publication bias for postoperative drainage

**Fig. 17:**
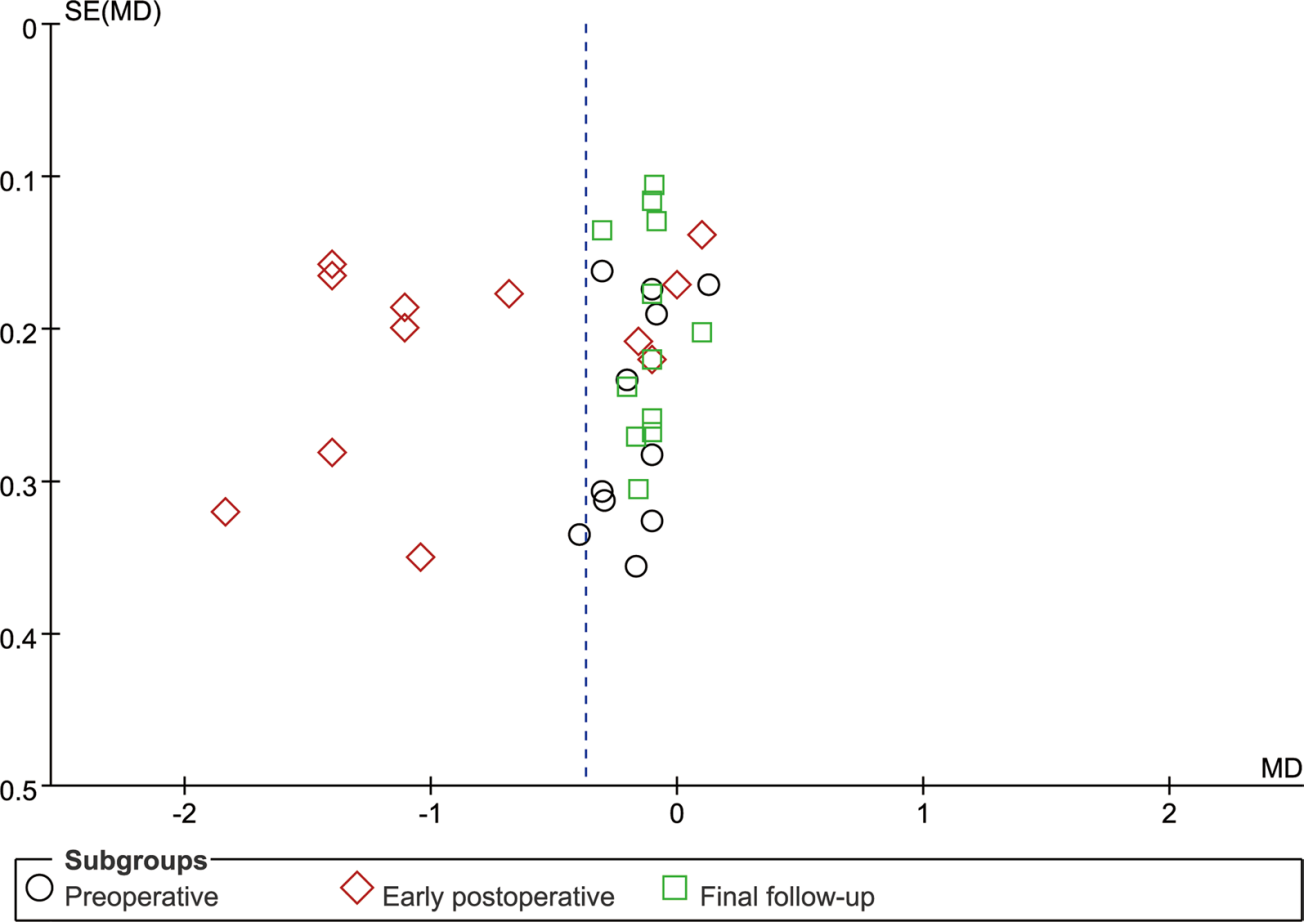
Funnel plot of publication bias for back VAS

**Fig. 18:**
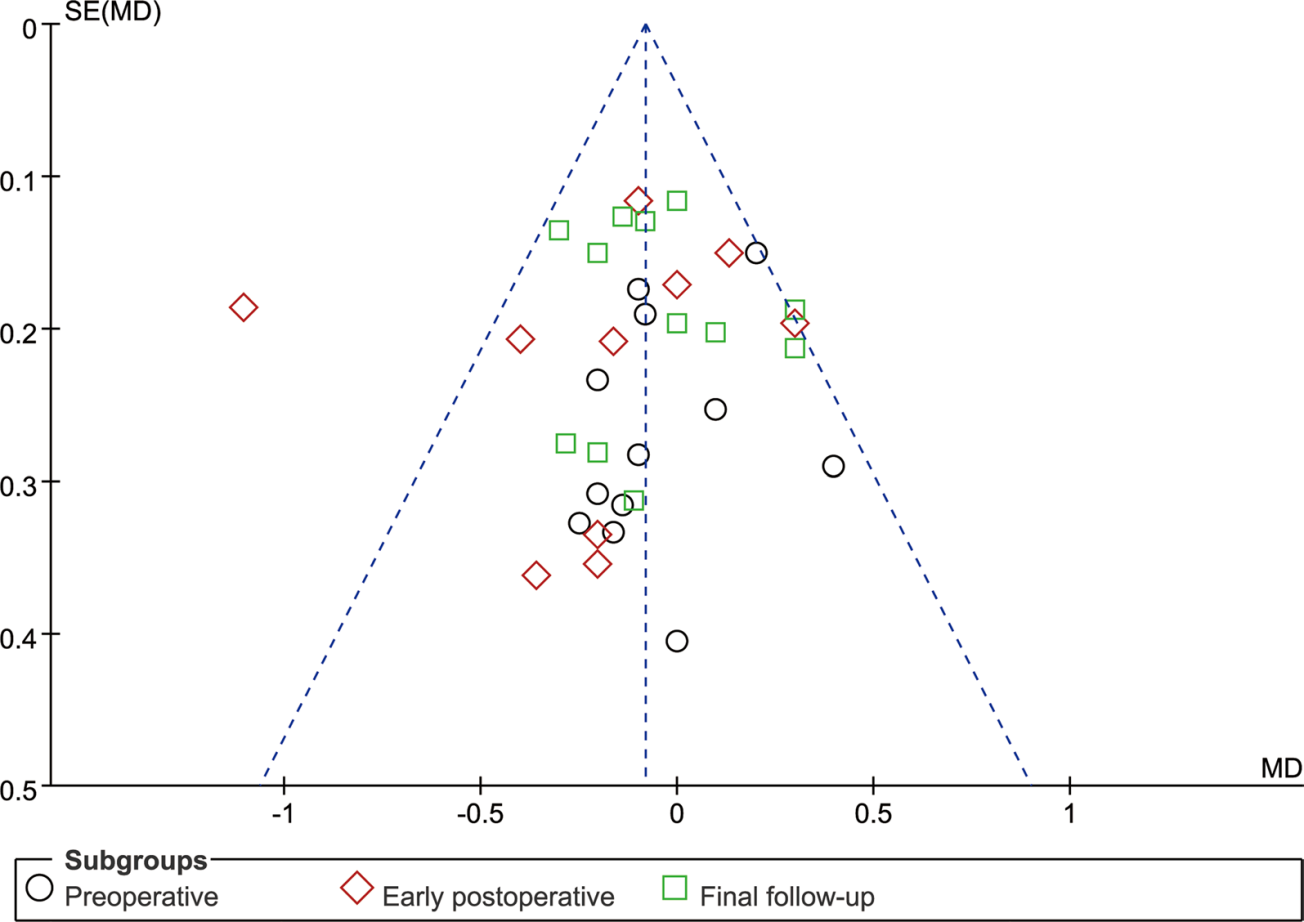
Funnel plot of publication bias for leg VAS

**Fig. 19:**
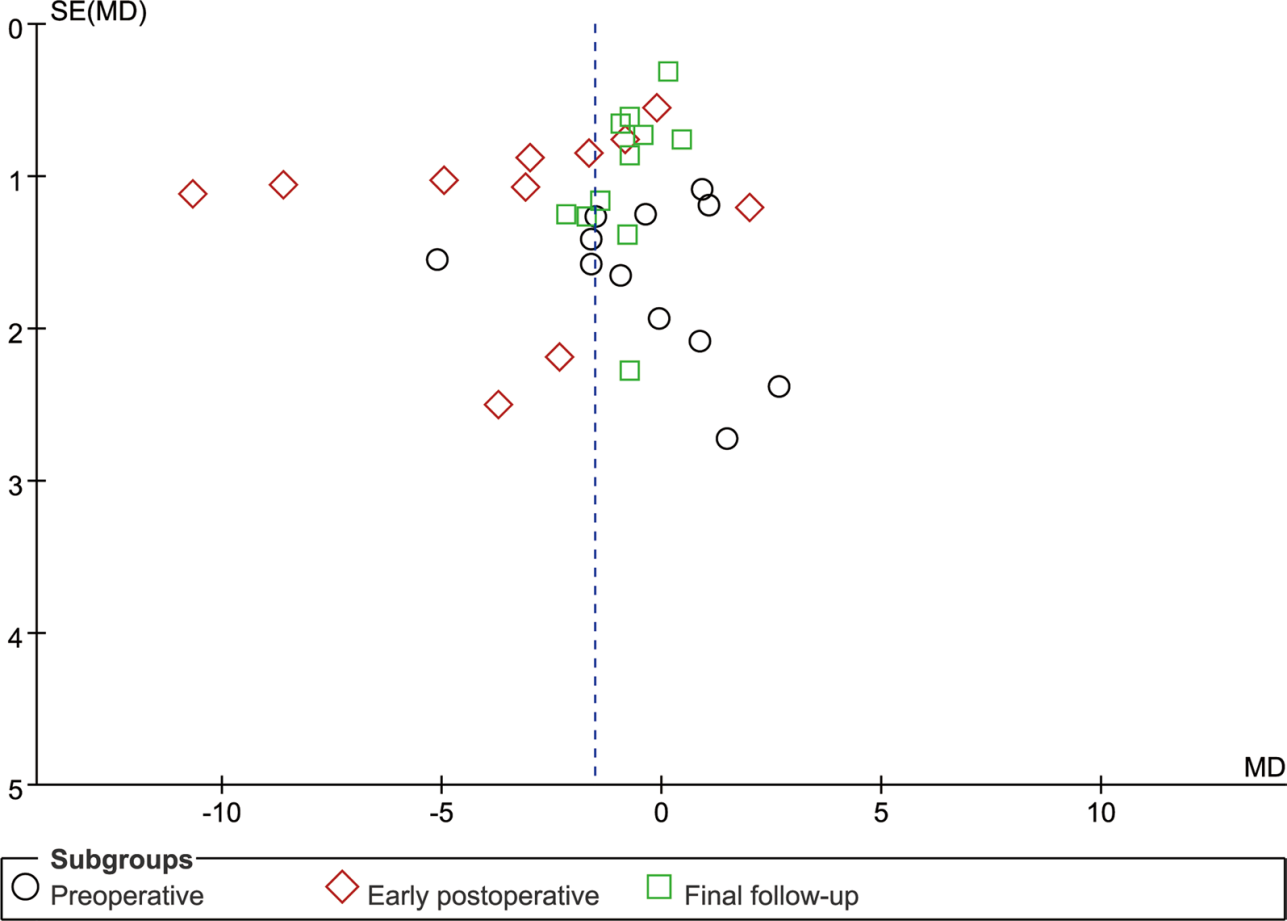
Funnel plot of publication bias for ODI

**Fig. 20:**
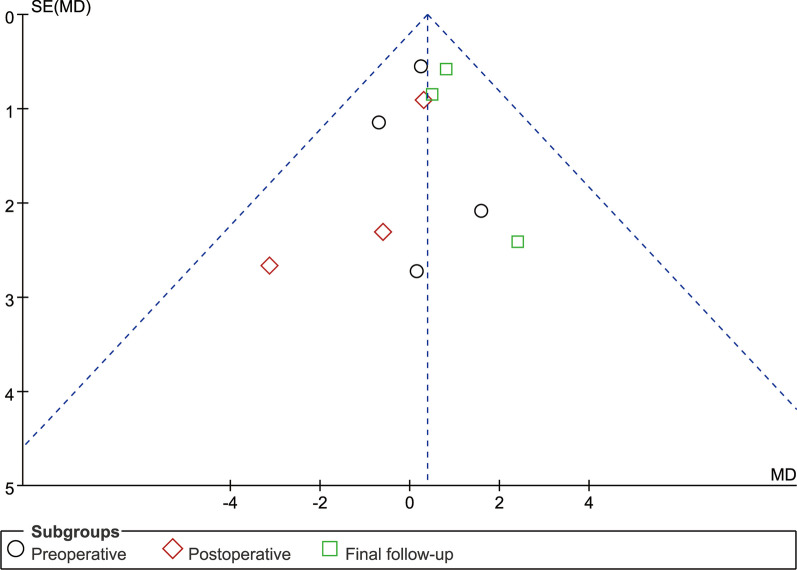
Funnel plot of publication bias for LL

**Fig. 21:**
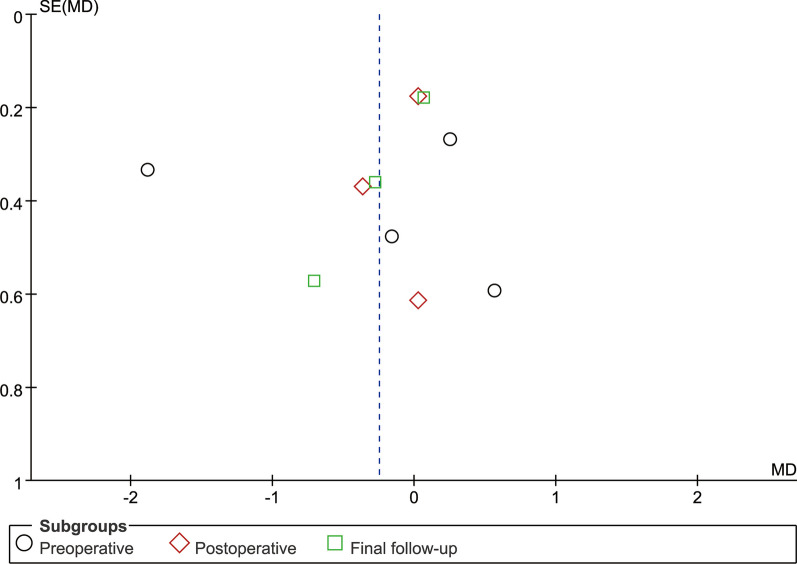
Funnel plot of publication bias for DH

**Fig. 22:**
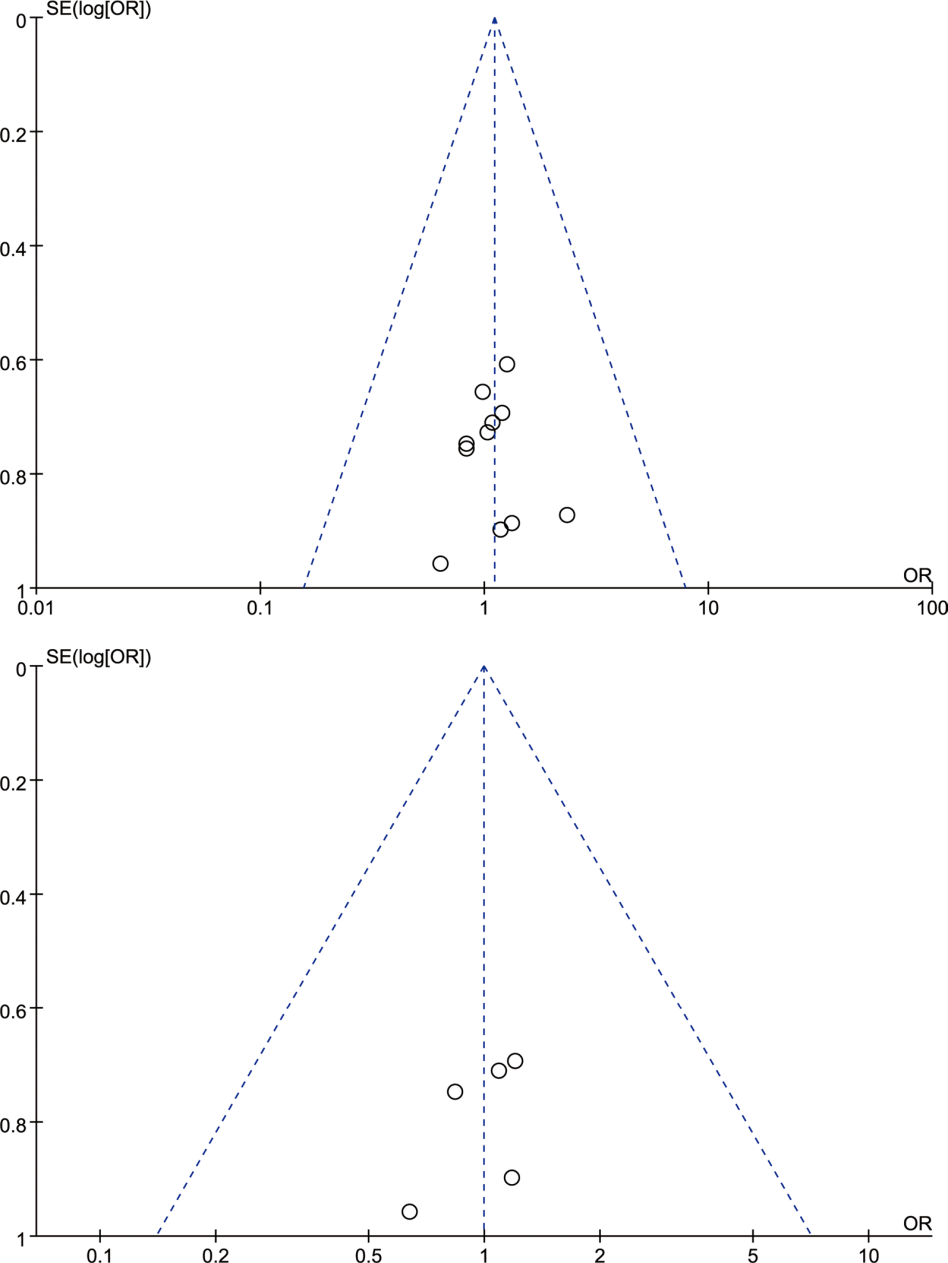
Funnel plot of publication bias for fusion rate

**Fig. 23:**
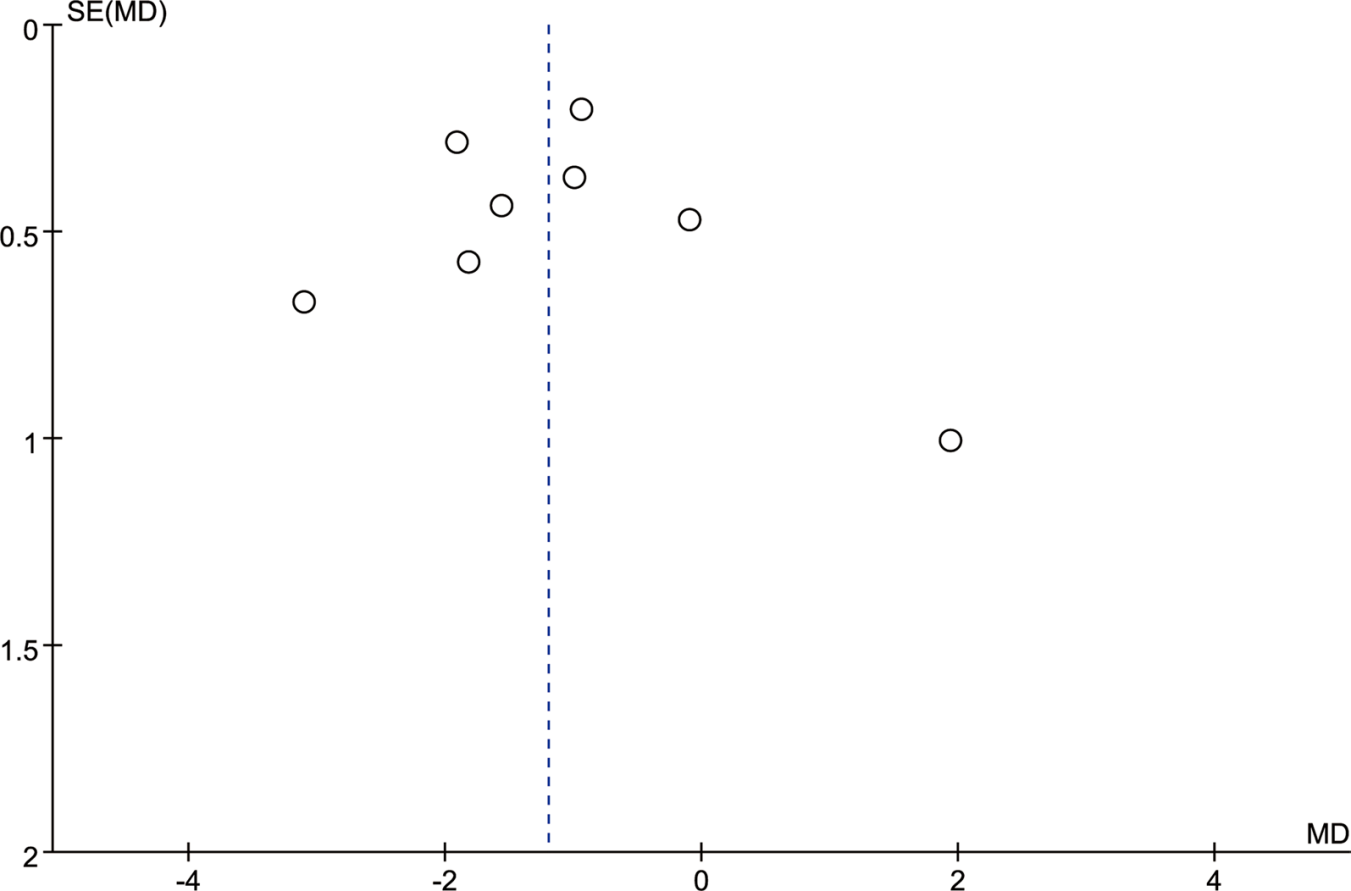
Funnel plot of publication bias for hospital length stay

**Fig. 24:**
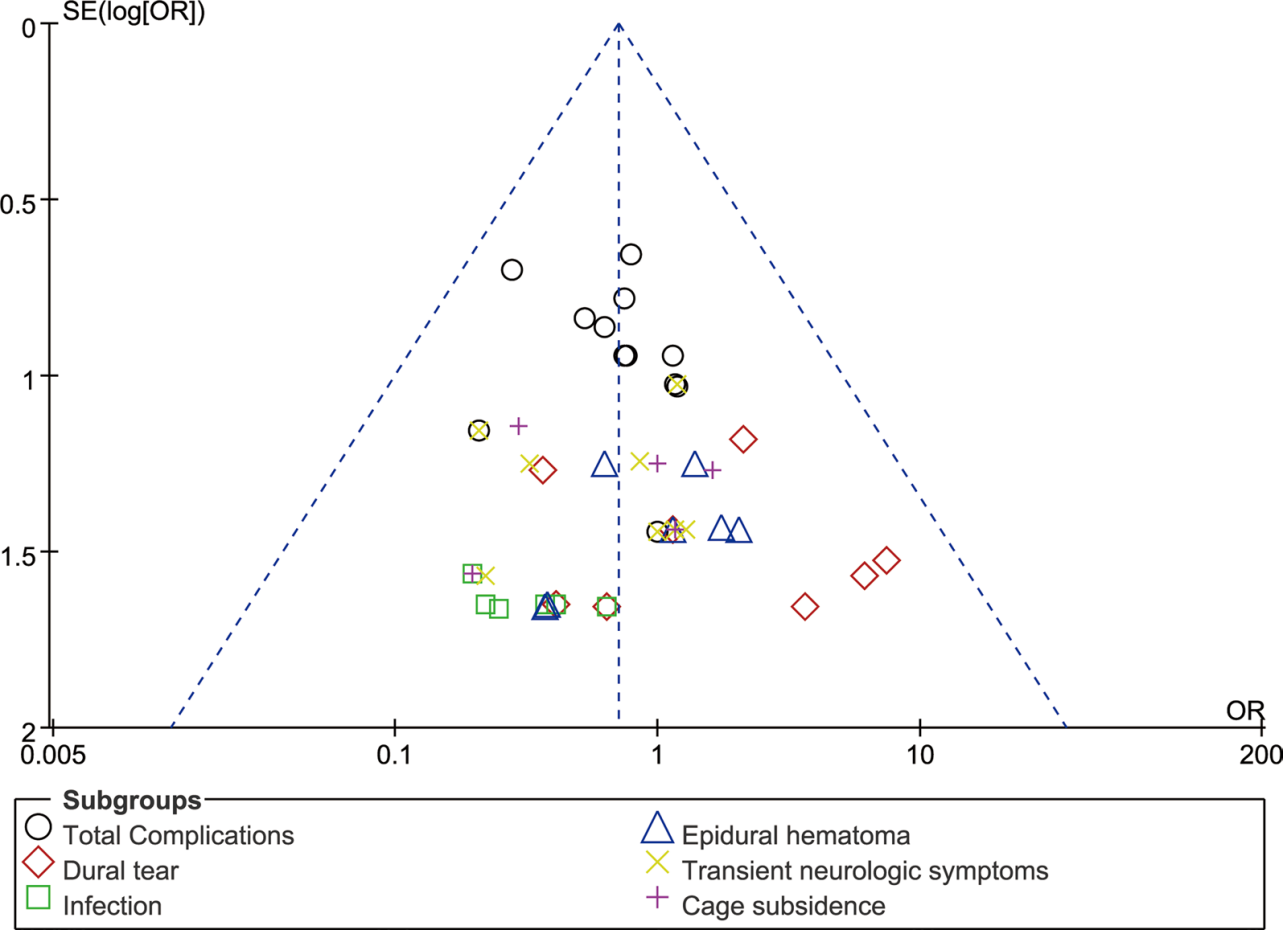
Funnel plot of publication bias for complications

## Discussion

With the rapid development of the concept, tools, and techniques of minimally invasive spinal surgery, how to make lumbar fusion surgery achieve the effect of decompression and fusion while minimizing trauma has become the goal pursued by spinal surgeons. MIS-TLIF employs tubular retractors and cold light source systems to selectively remove portions of the facet and lamina on the surgical side. By accessing the intervertebral disk through the transforaminal approach, it achieves nerve decompression and interbody fusion. Since its introduction to clinical practice, both orthopedic surgeons and patients have acknowledged MIS-TLIF for its precision and minimal invasiveness [25]. However, it still has the following disadvantages: (1) tubular retractors can also cause a certain degree of surgical trauma to the skin and muscle traction, which is easy to cause postoperative skin and muscle necrosis and long-term scar healing [26]; (2) the field of view using an air medium tends to blur easily, and the lighting in deeper channels and corners remains dim. This limitation becomes especially pronounced during contralateral undermining decompression; (3) endplate treatment depends on hand feeling, it is difficult to observe directly, and it is easy to have insufficient cartilage endplate treatment or damage the bony endplate [19].

The BE-TLIF technique is a new endoscopic technique that applies the traditional arthroscopic system to the spine, establishes an operating channel and an observation channel through a small incision, does not require an auxiliary tube to expose the field of view when completing spinal canal decompression and cage placement, has a large operating space, and is more flexible in the use of the device [5, 27]. BE-TLIF combines microscopic and endoscopic features and has the following advantages: (1) It has independent observation channels and working channels. By observing the channel perfusion arthroscope, the surgeon can directly look at the surgical field and clearly distinguish the structures around the nerve tissue; the working channel can be flexibly operated using conventional instruments for spinal surgery, with high work efficiency and easy popularization [3]. (2) Dissection of the paravertebral musculature rarely and maximally preserves the integrity of the spinal structure and maintains postoperative spinal stability; dissection of the paravertebral muscle close to the posterior laminar structure establishes a practical working space and is a familiar anatomy for the surgeon. (3) The procedure is conducted under continuous irrigation. Instead of a conventional electrotome, a radiofrequency electrode is used for hemostasis. This allows for effective cauterization of microvascular bleeding around the dural sac without causing nerve damage. Additionally, the absence of surgical smoke minimizes wound contamination, which can significantly reduce the risk of surgical site infections [8]. (4) UBE is not limited by a rigid conduit, the inner wall of the ipsilateral pedicle can be detected in the extent of decompression, and the contralateral side can reach the lateral recess by removing part of the spinous process root crossing the midline, and the dural sac, bilateral nerve root courses, and contralateral outlet root can be completely exposed after decompression to achieve fine exploration, release, and decompression of the nerve in the target area of the spinal canal under direct vision [15]. (5) The cartilage endplate can be completely removed and the bone graft bed can be prepared by magnifying the visual field under the microscope, which lays a good environment for bone graft fusion [3, 11].

The results of this study showed that the VAS score and ODI of low back pain and leg pain in the BE-TLIF group were lower than those in the MIS-TLIF group at the early postoperative follow-up, and the differences were statistically significant. The results showed that BE-TLIF could reduce the lumbar pain and improve the functional recovery of patients in the early stage. Because UBE was operated endoscopically throughout the operation, it had the advantages of both visual field magnification and flexible operation, which could protect the normal anatomy of the spine as much as possible and facilitate the early postoperative recovery [28]. This is consistent with Huang et al. [10] who also found that VAS score for lumbago and leg pain and ODI recovery in the early postoperative period were significantly better in the BE-TLIF group than in the MIS-TLIF group, and the length of hospital length stay was shorter, but there was no significant difference between groups at the final follow-up. Our findings indicate that the postoperative hospital length stay for the BE-TLIF group was shorter than that of the MIS-TLIF group, with the difference being statistically significant. This may be attributed to the more rapid alleviation of lumbar pain symptoms in patients who underwent BE-TLIF. As a result, these patients met discharge criteria sooner and were able to return to their normal lives more quickly.

In addition, compared with the MT-TLIF group, the BE-TLIF group had less intraoperative blood loss and postoperative drainage volume, and the reasons for less blood loss may be: (1) BE-TLIF requires normal saline irrigation fluid perfusion during surgical decompression and interbody bone grafting, has a certain water pressure, and plays a role in compression hemostasis; (2) BE-TLIF magnifies the surgical field through the imaging system, allowing for clear visualization of minor blood vessel bleeding on the monitor. By using a radiofrequency electrode for hemostasis, it achieves an effective bleeding control result [3]. However, the operation time was longer and the analysis may be related to the steep learning curve of this technique [29, 30]. Choi et al. [31] examined the learning curve of 68 UBE procedures conducted by a surgeon with 8 years of spinal surgery experience. They observed that the learning curve for UBE procedures began to stabilize after 36 cases. They emphasized that the clarity of the intraoperative visual field and effective bleeding control were crucial factors influencing the duration of the operation. A compromised intraoperative clarity could extend the time required to establish the workspace and identify surgical landmarks. Consequently, we advise spine surgeons to become proficient with the UBE technique and reach a stable point in the learning curve before undertaking the BE-TLIF procedure. This ensures that patients aren't negatively impacted by prolonged surgical durations.

In our study, there was no notable difference in the lumbar interbody fusion rate at the final follow-up between the two groups. This suggests that both BE-TLIF and MIS-TLIF procedures result in satisfactory fusion rates. While endplate bleeding serves as a reliable indicator, the preparation of the endplate remains crucial for successful interbody fusion [5, 32]. Because of the limited working space, bone bleeding can hardly be observed in procedures such as conventional MIS-TLIF. Surgeons generally estimate the completeness of preparation based on previous experience with the intervertebral space management; while BE-TLIF surgeons are able to directly observe the intervertebral space and remove the remaining annulus fibrosus and nucleus pulposus under direct vision [27]. LL and DH were significantly improved at the last follow-up in both groups compared with those before surgery, but there was no significant difference between the two groups, indicating that both BE-TLIF and MIS-TLIF could improve postoperative intervertebral stability and help to restore the normal sequence of the lumbar spine.

Common complications of BE-TLIF include dural tear, spinal epidural hematoma, inadequate decompression, iatrogenic instability, nerve root injury, infection, and postoperative numbness. Dural tears are a common complication of lumbar degenerative disease surgery, and studies have reported that the incidence of dural tears in lumbar degenerative disease surgery using BE-TLIF ranged from 2.9% to 5.8% [33]. Dural tear was considered to be due to severe stenosis of the spinal canal, and when bilateral decompression was performed by a unilateral approach, dural rupture was caused during dissection due to severe adhesion between the ligamentum flavum and the dura mater [34–36]. Kim et al. [37] concluded that even if dural tears occur, no management is needed because BE-TLIF is less damaging to the low back muscles and protects low back muscle function, so when dural tears occur, the low back muscles can play a role in preventing continuous leakage of cerebrospinal fluid. Spinal epidural hematoma after BE-TLIF is a rare complication among many complications after surgery, but due to its rapid progression, if not timely and effective treatment will cause devastating damage to spinal cord function. In terms of cage subsidence, it may be due to intraoperative destruction of the endplates. Therefore, when cleaning the endplate with more severe degeneration, do not be too violent to avoid damaging the endplate; secondly, when placing the interbody fusion cage, it should enter along the inclination angle of intervertebral space to prevent the interbody fusion cage from destroying the endplate, resulting in postoperative interbody fusion cage subsidence [18]. Complications such as nerve root injury, infection, and postoperative numbness are rarely reported. However, in this study, there was no significant difference in the overall complications and the incidence rate of the above complications between the two groups, and both were improved after symptomatic treatment.

The limitation of this study is that the included literatures are retrospective studies, there is no prospective study for reference, and there is a lack of long-term follow-up to comprehensively evaluate the safety and effectiveness of this technique. In addition, inconsistent follow-up times lead to differences in complications and fusion rates at the final follow-up. Therefore, subsequent prospective studies with large samples and multiple centers are needed to obtain higher levels of evidence support.

## Conclusion

In summary, BE-TLIF has the advantages of less intraoperative blood loss and postoperative drainage volume, rapid postoperative recovery, and effective protection of spinal soft tissues, and can achieve similar fusion rates and clinical effects as MIS-TLIF. Although the operation time is relatively long and has a relatively steep learning curve, it can be shortened by a certain accumulation of surgical volume. It provides a new option for minimally invasive treatment of lumbar degenerative diseases.

## Data Availability

The data sets generated and analyzed during the current study are not publicly available but can be obtained from the corresponding author on reasonable request.
